# Multi-tissue profiling of oxylipins reveal a conserved up-regulation of epoxide:diol ratio that associates with white adipose tissue inflammation and liver steatosis in obesity

**DOI:** 10.1016/j.ebiom.2024.105127

**Published:** 2024-04-26

**Authors:** Charlotte Hateley, Antoni Olona, Laura Halliday, Matthew L. Edin, Jeong-Hun Ko, Roberta Forlano, Ximena Terra, Fred B. Lih, Raúl Beltrán-Debón, Penelopi Manousou, Sanjay Purkayastha, Krishna Moorthy, Mark R. Thursz, Guodong Zhang, Robert D. Goldin, Darryl C. Zeldin, Enrico Petretto, Jacques Behmoaras

**Affiliations:** a Centre for Inflammatory Disease, Imperial College London, Hammersmith Hospital, Du Cane Road, London, W12 0NN, UK; b Centre for Computational Biology and Program in Cardiovascular and Metabolic Disorders, Duke-NUS Medical School, Singapore, Singapore; c Department of Surgery and Cancer, Imperial College London, UK; d Division of Intramural Research, NIEHS/NIH, Research Triangle Park, NC, USA; e Division of Brain Sciences, Imperial College Faculty of Medicine, London, UK; f Department of Metabolism, Digestion and Reproduction, Imperial College London, UK; g Imperial College Healthcare NHS Trust, St. Mary's Hospital, Praed Street, London, W2 1NY, UK; h Universitat Rovira i Virgili, Departament de Bioquímica i Biotecnologia, MoBioFood Research Group, Tarragona, Spain; i Department of Nutrition, College of Agriculture and Environmental Sciences, 3135 Meyer Hall, One Shields Avenue, UC Davis, Davis, CA, 95616, USA; j Institute for Big Data and Artificial Intelligence in Medicine, School of Science, China Pharmaceutical University (CPU), Nanjing, China; k University of Brunel, Kingston Lane, Uxbridge, London, UB8 3PH, UK

**Keywords:** Obesity, Metabolic syndrome, Oxylipins, Epoxides, Diols, 12,13-EpOME:DiHOME

## Abstract

**Background:**

Obesity drives maladaptive changes in the white adipose tissue (WAT) which can progressively cause insulin resistance, type 2 diabetes mellitus (T2DM) and metabolic dysfunction-associated liver disease (MASLD). Obesity-mediated loss of WAT homeostasis can trigger liver steatosis through dysregulated lipid pathways such as those related to polyunsaturated fatty acid (PUFA)-derived oxylipins. However, the exact relationship between oxylipins and metabolic syndrome remains elusive and cross-tissue dynamics of oxylipins are ill-defined.

**Methods:**

We quantified PUFA-related oxylipin species in the omental WAT, liver biopsies and plasma of 88 patients undergoing bariatric surgery (female N = 79) and 9 patients (female N = 4) undergoing upper gastrointestinal surgery, using UPLC-MS/MS. We integrated oxylipin abundance with WAT phenotypes (adipogenesis, adipocyte hypertrophy, macrophage infiltration, type I and VI collagen remodelling) and the severity of MASLD (steatosis, inflammation, fibrosis) quantified in each biopsy. The integrative analysis was subjected to (i) adjustment for known risk factors and, (ii) control for potential drug-effects through UPLC-MS/MS analysis of metformin-treated fat explants *ex vivo*.

**Findings:**

We reveal a generalized down-regulation of cytochrome P450 (CYP)-derived diols during obesity conserved between the WAT and plasma. Notably, epoxide:diol ratio, indicative of soluble epoxide hydrolyse (sEH) activity, increases with WAT inflammation/fibrosis, hepatic steatosis and T2DM. Increased 12,13-EpOME:DiHOME in WAT and liver is a marker of worsening metabolic syndrome in patients with obesity.

**Interpretation:**

These findings suggest a dampened sEH activity and a possible role of fatty acid diols during metabolic syndrome in major metabolic organs such as WAT and liver. They also have implications in view of the clinical trials based on sEH inhibition for metabolic syndrome.

**Funding:**

10.13039/100010269Wellcome Trust (PS3431_WMIH); 10.13039/100016017Duke-NUS (Intramural Goh Cardiovascular Research Award (Duke-NUS-GCR/2022/0020); 10.13039/501100001349National Medical Research Council (OFLCG22may-0011); 10.13039/100000066National Institute of Environmental Health Sciences (Z01 ES025034); 10.13039/501100013342NIHR Imperial Biomedical Research Centre.

**Research in context**

**Evidence before this study**

The crosstalk between the white adipose tissue (WAT) and the liver is a central feature of obesity and metabolic syndrome. Oxylipins were studied in association with metabolic syndrome in human patients but the specific involvement of Cytochrome P450–soluble epoxide hydrolase (CYP-sEH) pathway remains controversial due to study designs restricted to systemic associations and/or usage of inadequate animal models.

**Added value of this study**

Here we describe cross-tissue dynamics of oxylipins in the context of evolving visceral WAT and liver pathologies in patients with obesity undergoing bariatric surgery.

• We use an integrative, multi-tissue approach, focussing on conserved oxylipin dynamics between WAT and plasma, following data-driven and experimental (*ex vivo* culture-based) filtering approaches.
• We reveal significant changes in CYP-sEH pathway in the WAT, liver and plasma of individuals with metabolic syndrome.
• CYP-derived diols, whether AA, LA or DHA-derived, are reduced in WAT and plasma of patients with obesity when compared to lean controls.
• Epoxide:diol ratio, a surrogate of soluble epoxide hydrolyse (sEH) activity, increases with WAT inflammation, hepatic steatosis and T2DM.
• Increased 12,13-EpOME:DiHOME in WAT and liver is a marker of worsening metabolic syndrome in patients with obesity.

**Implications of all the available evidence**

Our findings demonstrate a defective CYP-sEH pathway during obesity that should be taken into account for designing clinical trials based on sEH inhibition to target the metabolic complications of obesity. These results suggest an overall reduced WAT and liver sEH activity during metabolic syndrome. They also highlight a previously unappreciated role of fatty acid diols in adipose tissue homeostasis.

## Introduction

Obesity is strongly associated with metabolic syndrome and is linked to the impairment of glucose and lipid metabolism which can result in type 2 diabetes mellitus (T2DM) and metabolic dysfunction-associated liver disease (MASLD).^1^ When there is prolonged overnutrition, the white adipose tissue (WAT) becomes progressively inflamed and dysfunctional.^2^, ^3^, ^4^ This maladaptive state of the WAT can cause surplus lipid release and deposition within the liver and other tissues,^5^ potentiating insufficient insulin secretion from pancreatic β-islet cells, insulin resistance and development of T2DM.^3^^,^^6^^,^^7^ MASLD affects up to 96% of patients with obesity, though only a proportion of these patients will develop steatohepatitis (7–35%)^8^ as result of hepatic inflammation and lipid deposition.

When caloric excess occurs, WAT expands its mass through adipocyte hypertrophy and/or by recruitment of new adipocytes. The latter is known as hyperplasia^9^ and involves *de novo* adipocyte differentiation (i.e. adipogenesis) from precursor cells^10^ which depend on mitochondrial bioenergetics^11^ and a fine balance between lipogenic and lipid-scavenging activities.^12^ Adipogenesis, recently proposed as a regenerative cycle of adipocyte death and birth,^13^ is central to healthy WAT regeneration and expansion, and is regulated by concerted actions of key transcription factors such as peroxisome proliferator-activated receptor gamma (PPARγ) and CCAAT/enhancer-binding protein alpha (CEBPα).^3^^,^^14^ Concomitant with adipocyte hypertrophy and hyperplasia, other hallmarks of prolonged obesity include unresolved low-grade macrophage-mediated inflammation, changes in vascularization and hypoxia-mediated dysregulation of extracellular matrix (ECM) remodelling in the WAT.^2^^,^^15^, ^16^, ^17^, ^18^

Polyunsaturated fatty acid (PUFA) metabolism is central to adipose tissue health. PUFAs such as arachidonic acid (AA), linoleic acid (LA), eicosapentaenoic acid (EPA) and docosahexaenoic acid (DHA) reside in cell membrane phospholipids or can be stored within triacylglycerols (TGs). PUFA-containing TGs increase during obesity in human WAT^19^ and during pro-inflammatory macrophage activation.^20^ Upon cell activation, PUFAs are released and subsequently oxidized by three sets of distinct enzymes [lipoxygenases (LOX), cyclooxygenases (COX) and cytochrome P450 (CYP) family enzymes—see detailed reviews^21^^,^^22^] to produce a variety of bioactive oxidized lipids known as oxylipins, a superfamily that comprise of eicosanoids (i.e. lipid mediators derived from 20-carbon PUFAs). These short-lived soluble lipids can control obesity-induced WAT adaptations at almost every level: fundamentally, oxylipins orchestrate lipid metabolism and adipocyte differentiation through binding to nuclear transcription factors and regulating their activity.^23^^,^^24^ Among these transcription factors, peroxisome proliferator-activated receptors (PPARs),^25^, ^26^, ^27^, ^28^, ^29^, ^30^ including PPARγ, mediate the maturation of adipocytes.^31^^,^^32^ On the other hand, oxylipins accompany immune homeostasis and tissue inflammation. They control tissue-resident macrophage maintenance during homeostasis^33^ and can be secreted or used in mitochondrial β-oxidation during the pro-inflammatory activation of macrophages.^21^^,^^34^ In the vasculature, oxylipins that include CYP-derived fatty acid epoxides were identified as endothelium-derived hyperpolarizing factors^35^^,^^36^ that can exert angiogenic properties.^37^^,^^38^ Since metabolic, adipogenic, immune (macrophage-mediated) and vascular/angiogenic pathways define the WAT remodelling during obesity,^3^^,^^10^ oxylipin networks are part of the endocrine function of the WAT and are likely to regulate its crosstalk with other metabolic organs such as the liver. The crosstalk between the adipose tissue and the liver being an important feature of obesity-mediated T2DM and MASLD, tissue-specific oxylipin signatures and their association with local stress can provide a more accurate understanding of the role of these bioactive lipid pathways during metabolic syndrome.

Oxylipins were studied in association with obesity/T2DM or MASLD/Metabolic dysfunction-associated steatohepatitis (MASH) in human patients.^39^, ^40^, ^41^, ^42^, ^43^, ^44^, ^45^, ^46^, ^47^, ^48^, ^49^, ^50^, ^51^, ^52^, ^53^, ^54^, ^55^, ^56^, ^57^ COX and LOX-derived oxylipins were generally found to increase during metabolic syndrome,^41^^,^^46^^,^^48^^,^^54^, ^55^, ^56^, ^57^ though the association of CYP-derived epoxides and their corresponding diols remains unclear. While most studies used patient plasma for epoxide/diol associations, one study reported changes in subcutaneous WAT,^43^ whose expansion is a lesser risk for metabolic syndrome than visceral WAT.^58^, ^59^, ^60^ However, to date, there is no study describing cross-tissue oxylipin dynamics in the context of evolving visceral WAT and liver phenotypes during obesity, T2DM and MASLD.

Here we first present a detailed phenotyping of the omental WAT isolated from a bariatric surgery cohort. We then quantified 5 PUFAs and 71 PUFA-related oxylipin species in WAT, liver and plasma and correlated their abundance with WAT phenotypes (adipogenesis, adipocyte hypertrophy, macrophage infiltration, fibrosis) and histopathological severity of MASLD (NAS score or percentage of ballooning, fat, inflammation, and collagen quantified in each biopsy). We reveal significant changes in the CYP epoxide-diol pathway with a conserved up-regulation of epoxide:diol that accompanies WAT inflammation and liver steatosis during obesity-mediated metabolic syndrome.

## Methods

### Patient recruitment and sample collection

Patients with obesity undergoing bariatric surgery and lean control patients undergoing upper gastrointestinal surgery for local oesophageal or gastric carcinoma were recruited and informed consent was obtained for involvement in this project. The lean samples were selected based on (i) comparable anatomical location of visceral fat to those undergoing bariatric surgery, (ii) evidence of no metastatic disease, and (iii) the surgery site being distant from visceral fat. Participants were excluded from the study if they were on insulin, immunosuppressive medication or medication that can cause hepatic steatosis. They were also excluded if they had a blood-borne virus, autoimmune disease or active systemic malignancy. All patients were over the age of 18; obese or morbidly patients with obesity were included with BMI >30 and lean controls with BMI 18–25. Demographic and clinical data were collected from routine clinical tests available. Sex and ethnicity were self-reported.

Omental white adipose tissue and liver tissues were collected at the time of bariatric or upper gastrointestinal surgery. 1 g of adipose tissue was snap-frozen, 0.5–1 g was fixed in 10% formalin for immunohistochemistry (IHC) analysis. A further 6 g of adipose tissue sample was kept on ice and immediately used for stromal vascular fraction (SVF) isolation or for *ex vivo* explant culture experiments. A subcapsular liver biopsy was dissected, and a cross-sectional piece was fixed in 10% formalin for histology, and a further 40 mg piece was snap-frozen. 10 ml of whole blood was collected in EDTA-vacutainer for plasma isolation. Whole blood in EDTA vacutainer (BD Vacutainer™, Cat#VS367873) was centrifuged and plasma was retained for oxylipin ultra-performance liquid chromatography-mass spectrometry (UPLC-MS/MS) analysis (See also Figure S1 for study design).

### Stromal vascular fraction (SVF) isolation

White adipose tissue collected from patients undergoing bariatric surgery was cut into fine pieces and incubated at 37 °C for 50–60 min in adipose tissue digestion buffer that contains HBSS, 0.5% BSA, 10 mM CaCl_2_ and 4 mg/ml collagenase type I (Sigma–Aldrich; Cat#C0130). Once the tissue was completely digested, the suspension was then passed through a 200 μM strainer (Pluriselect; Cat#43-50200-01) and centrifuged at 300 g for 10 min at 4 °C. The adipose layer was removed and the SVF was resuspended in red cell lysis buffer (Sigma–Aldrich; Cat#R7757) on ice for 3 min. Following further centrifugation at 300*g* for 10 min, the SVF was used for flow cytometry analysis.

### Flow cytometry

Following SVF isolation from the whole WAT, the pellet enriched for the SVF is resuspended and used either for monocyte/macrophage detection and quantification or for assessing cell viability in response to treatment with metformin. Monocyte/macrophage detection and quantification was assessed by staining with commercially validated antibodies CD14-PE (BioLegend; Cat#301806, clone:M5E2), CD45-FITC (BioLegend; Cat#304005, clone: HI30), CD206-BV-421 (BioLegend; Cat#321125, clone: 15-2), CD3-APC (BioLegend; Cat#317318, clone: OKT3). All antibodies were diluted 1:100 in 100 μl FACS buffer (1% BSA, 5 mM EDTA, 0.05% Sodium Azide in PBS) and incubated at 4 °C, for 30 min. SVF was then centrifuged at 300 g for 5 min and resuspended in Dead/Live stain diluted 1:200 (Thermo Fisher Scientfic; Cat#L34975) and incubated for further 10 min on ice. Cells were then centrifuged at 300 g for 5 min and fixed in 1% PFA before flow cytometry. For measuring cell viability, SVF was stained with APC- Annexin V (1:30 dilution) and propidium iodide (PI; 1:50 dilution) according to the manufacturer's instructions (BioLegend; Cat#79998). All samples were acquired on a LSRFortessa flow cytometer (BD Biosciences, USA) and data were analysed with FlowJo software, version 10 (Tree Star Inc. Ashland, OR, USA).

### Ex vivo white adipose tissue culture

Whole adipose tissue was collected at the time of bariatric surgery, weighed and immediately divided into 150 mg pieces under sterile conditions. Samples were incubated at 37 °C 5% CO_2_ with 3 ml DMEM (Gibco; Cat#4196S-039) enriched with 10% FBS and 1% penicillin-streptomycin (Sigma–Aldrich; Cat# P4333). For metformin-treated explants, the culture media was supplemented with metformin (Sigma–Aldrich; Cat#317240) reconstituted in water at indicated concentrations. Explants were incubated for 48 h and harvested for SVF isolation (measurement of the metformin effect on cell viability) or for UPLC-MS/MS.

### Quantitative Reverse Transcription PCR (qRT-PCR)

qRT-PCR was performed on whole WAT, and liver tissue. Whole adipose tissue or liver tissues were suspended in 1 ml Trizol (Ambion Life Technology; Cat#15596026) and total RNA was extracted using mechanical homogenization. 200 μl chloroform was then added to the solution and incubated at room temperature for 5 min. Followed by centrifugation at 12,000 rpm for 20 min. Total RNA was extracted using RNA extraction kit (Qiagen; Cat#74106) according to the manufacturer's instructions. Complementary DNA (cDNA) was obtained using the Bio-Rad iScript kit (Bio-Rad, UK; Cat#1708891) and quantitative Reverse Transcription PCR (qRT-PCR) reactions were performed using the ViiA 7 Real-Time PCR system (Life Technologies). A total of 5 ng of cDNA per sample was used for each reaction, using Brilliant II SYBR Green QPCR Master Mix (Agilent; Cat#600828). ViiA 7 RUO Software was used for the determination of Ct values and results were analysed using the comparative Ct method with each WAT or liver sample normalised to Actin or HPRT genes. Primers sequences used are listed in Table S1.

### Oxylipin quantification

Metabolites were extracted from omental WAT, liver and plasma samples by solid-phase extraction and quantified by UPLC-MS/MS similar to what was previously described.^61^

#### Omental WAT

Frozen omental WAT (50 mg) was homogenized in 350 μl ice-cold methanol by shaking with a steel bead for 5 min at 30 hz. Samples were centrifuged at 1000×*g* for 5 min at 4 °C, and supernatants were mixed with 10 μl of internal standards [(PGE2-d9 (300 ng), LTB4-d4 (300 ng), 11,12-DHET-d11 (150 ng), 11,12-EET-d11 (300 ng), 15-HETE-d8 (300 ng), AA-d11 (750 ng)]. The recovery of each was PGE2-d9 (300 ng), LTB4-d4 (300 ng), 11,12-DHET-d11 (150 ng), 11,12-EET-d11 (300 ng), 15-HETE-d8 (300 ng), AA-d11 (750 ng). The supernatants were then combined with 2.7 ml of water and passed through HyperSep Retain SPE columns (Thermo Fisher Scientific, Bellefonte, PA). The columns were washed with 2.5% methanol, 0.05% acetic acid, and then eluted with 1 ml of methanol and 1 ml of ethyl acetate into glass tubes containing 6 μl of 30% glycerol in methanol. The eluates were dried under vacuum centrifugation and reconstituted in 50 μl of 30% ethanol.

#### Plasma

For quantification of free/non-esterified plasma oxylipins, 150 μl of plasma was mixed with 450 μl methanol and 10 μl of an internal standard solution (PGE2-d9 (300 ng), LTB4-d4 (300 ng), 11,12-DHET-d11 (150 ng), 11,12-EET-d11 (300 ng), 15-HETE-d8 (300 ng), AA-d11 (750 ng). The recovery of each was PGE2-d9 (300 ng), LTB4-d4 (300 ng), 11,12-DHET-d11 (150 ng), 11,12-EET-d11 (300 ng), 15-HETE-d8 (300 ng), AA-d11 (750 ng). Samples were then mixed and centrifuged at 10,000 g for 10 min. The supernatant was transferred to an Eppendorf tube and dried under vacuum centrifugation. Samples were reconstituted in 800 μl ethyl acetate and passed through a phospholipid removal column (Cerex Maestro, 15 mg, Tecan). The column was washed with 500 μl acetonitrile and dried under vacuum centrifugation.

#### Liver

For quantification of free/non-esterified liver oxylipins, frozen liver biopsies were weighed and placed in a tube with 19 μl HBSS/mg liver. Each tube received 1 μl of 1 mM TPPU in methanol. Liver tissue was homogenized using a Tissuelyzer II (Qiagen) set to 30 Hz for 10 min. 10 μl internal standard mix (PGE2-d9 (300 ng), LTB4-d4 (300 ng), 11,12-DHET-d11 (150 ng), 11,12-EET-d11 (300 ng), 15-HETE-d8 (300 ng), AA-d11 (750 ng) with 100% recovery, was added to 100 μl (5 mg) of liver tissue and boiled for 5 min. Free oxylipins were immediately extracted by liquid:liquid extraction with 800 μl ethyl acetate and dried under vacuum centrifugation. To determine “total” oxylipins (free and sn-2 esterified), a separate 5 mg of lysate was incubated with 3 Units of porcine pancreas PLA2 (Sigma–Aldrich; Cat#6534) to hydrolyze sn-2-esterified oxylipins before liquid:liquid extraction.

Online chromatography of oxylipins was assayed on an Utimate 3000 UHPLC equipped with an Xselect CSH C18, 2.1 × 50 mm, 3.5 μm particle column (Waters, Wilford, MA) and a TSQ Quantiva tandem mass spectrometer (Thermo Fisher Scientific, Waltham, MA). Quantification was determined using multiple reaction monitoring, and quantified by a blinded observer using TraceFinder (v4.1, Thermo Fisher Scientific). Peaks for analytes with less than ∼10 signal to noise were scored as below detection. For each sample and analyte, a relative response ratio (peak area of analyte/peak area of recovered internal standard (ISTD)) was determined and compared to a standard curve of relative response ratios of known concentrations of standards.^62^ As a measure of data quality, we have listed the average signal-to-noise ratios for each analyte for both adipose tissue and liver (free; Table S2). To ensure that our quantifications (used in the statistical analysis) are accurate and reproducible, we ran WAT samples (n = 84) twice and found that 88% of the metabolites correlated significantly between the 2 run (p < 0.05).

71 oxylipins were measured in plasma, WAT and liver samples. Samples were excluded from the analysis if they were under the detection limit. Metabolites undetectable in more than 50% of the samples were not included in the final analysis. Amongst those, 19, 24 and 27 were undetectable in liver, WAT and plasma, respectively. A further 2 samples were excluded from the liver and plasma analysis.

### WAT immunohistochemistry and adipocyte area quantification

For CD68 immunostaining, 5 μm thick paraffin WAT sections were dewaxed and rehydrated. Antigen retrieval with sodium citrate buffer (pH 6) was carried out prior to blocking. The sections were then incubated with primary antibodies against CD68 (Bio-Rad 1:200; Cat#MCA5709, clone KP1), washed in PBS, further incubated with horseradish peroxidase-labelled secondary antibody (1:10,000) for 1 h at room temperature (DAKO EnVision™+ System; Agilent Technologies (UK); Cat#4065). Diaminobenzidine (DAB) chromogen was then added, and the slides were visualized using brightfield images taken on the Leica DM4B microscope.

Haemotoxylin and eosin staining was performed as part of routine immunohistochemistry service provided by Imperial College London. Adipocyte mean areas were measured using 10X brightfield images taken on the Leica DM4B microscope. They were analysed using Image J Adiposoft plug-in.^63^ The scale was set using a scale bar and the minimum and maximum areas were set to 20 and 20,000 μm^2^, respectively. Of note, the median size of adipocytes in the obese cohort was 3400 μm^2^; mean adipocyte area above and below this threshold was considered large and small, respectively.

For immunofluorescence, 5 μm thick paraffin sections were dewaxed and rehydrated. Antigen retrieval with sodium citrate buffer (pH 6) was carried out prior to blocking. Slides were then incubated overnight with Anti rabbit -Type VI Collagen (Abcam 1:200; Cat#ab199720) or Anti goat-collagen I (Southern Biotech, 1:100; Cat#1310-01). After washing, slides were incubated for 1 h with Donkey Anti-Goat AlexaFluor555 (Abcam 1:400; Cat#ab150130) or Donkey anti-rabbit AlexaFluor555 (Invitrogen 1:100; Cat#A-31572). Slides were then mounted using VECTASHIELD with DAPI (Abcam; Cat#H-1200-10) medium. Images were taken using epi-fluorescent Leica DM4B microscope and mean grey values acquired using ImageJ software.

### Western blotting

Protein lysates from whole white adipose tissue were homogenized in RIPA buffer (Thermo Fisher Scientific; Cat#89901) (25 mM Tris–HCl pH 7.6, 150 mM NaCl, 1% NP-40, 1% sodium deoxycholate, 0.1% SDS) supplemented with 1% protease inhibitor Cocktail (Thermo Fisher Scientific; Cat#78430) and 1% phosphatase inhibitors (Cell Signaling Technology; Cat#5870 S) and incubated at 4 °C for 2 h. Lysates were then centrifuged for 20 min at 12,000 g, at 4 °C and supernatants were used for Western blot analysis. Total protein concentration is determined by Bicinchoninic Acid Kit for Protein Determination (Thermo Fisher Scientific; Cat#23227). Final protein lysates were resolved by SDS-PAGE 10% at 200 V for 1 h and transferred to PVDF membranes in 20% methanol at 300 mA for 1 h. The membrane was blocked for 1 h at room temperature using 5% BSA or 5% skimmed milk and then incubated overnight at 4 °C with primary antibodies. The blots were exposed to horseradish peroxidase-labeled secondary antibody for 1 h at room temperature. The immunocomplexes were visualized by the chemiluminescence detection system SuperSignal West Pico PLUS Substrate (Thermo Fisher Scientific; Cat#34580). Primary antibodies used were anti-PPARγ (Cell Signaling Technology 1:1000; Cat#C26H12), anti-CEBPα (Cell Signaling Technology 1:1000; Cat#2295 S) and anti-actin-b (Santa Cruz 1:12,000; Cat#SC-47778). Secondary antibodies used were rabbit anti-mouse antibody (Dako 1:6000; Cat#P0260) or swine anti-rabbit antibody (Dako 1:5000; Cat#P0217). All antibodies were commercially validated (certificates included in Supplementary).

### Liver histopathology and NAFLD Activity Score CRN

Haematoxylin and eosin and Sirius Red staining of liver samples were performed as part of immunohistochemistry service provided by Imperial College London. Liver histopathology scoring for MASLD was performed using NAFLD Activity Score CRN and fibrosis score system.^64^ This was used to stratify the patients into 3 groups (0–2 = no MASH, 3–4 = Intermediate (MASLD/MASH) and 5+ = MASH). Liver biopsies were also scored using a verified software package that analysed haematoxylin and eosin and Sirius red staining for histologic features of MASLD by quantifying (percentage) collagen deposition (collagen proportionate area; CPA), steatosis, ballooning and inflammation for each specimen.^65^

### Ethics

Patients with obesity undergoing bariatric surgery and lean control patients undergoing upper gastrointestinal surgery at St Mary's and Hammersmith Hospitals, Imperial College Healthcare NHS Trust, London between June 2018 and August 2021, were recruited under the ethical and Health Research Authority (HRA) approval (REC 19/WM/0229). Informed consent was obtained from all participants involved and the study complied with ethical guidance.

### Statistics

Results were analysed using GraphPad Prism 9 software (GraphPad, USA) or IBM SPSS statistics UK version 29.0.0.0 (241). All data are expressed as the mean ± SEM. Firstly, normality of the data was tested using Kolmogorov–Smirnov and the Shapiro–Wilk tests.

#### Univariate analysis

Statistical analyses for normally distributed grouped data were performed using parametric statistics either using student's t-test (two-way) or one-way Analysis of variance (ANOVA).

For non-normally distributed grouped data, we used non-parametric statistics; Mann–Whitney U test or Kruskal–Wallis test, applying appropriate post-hoc tests.

**Table 1:** Clinical characteristics and metabolic profiling of lean and patients with obesity undergoing bariatric surgery stratified into T2DM (HbA1C ≥ 48 mmol/mol or clinical diagnosis documented) and non-T2DM (HbA1C < 48 mmol/mol or no clinical diagnosis).

	Lean (n = 9)	Obese (n = 61)	Obese + T2DM (n = 27)	p-value
**Age**	61.1 ± 13.4	47.08 ± 13.71	52.96 ± 8.7	0.01
**Sex** (F)	4	55	24	0.005
**Ethnicity**				0.74
Asian/British Asian	1	6	4	
Black/Black Caribbean	0	5	2	
White	3	27	16	
Other	5	14	6	
Mixed	0	2	0	
**BMI** (m^2^/kg)	22.9 ± 3.7	45.78 ± 7.6	48.15 ± 7.8	<0.0001
**HbA1C** (mmol/moll)	39.7 ± 5.0	37.64 ± 4.5	60.33 ± 18.1	<0.0001
**Cholesterol** (mg/dL)	4.5 ± 1.3	4.8 ± 0.99	4.57 ± 1.09	0.43
**Triglyceride** (mg/dL)	1.2 ± 0.68	1.8 ± 2.4	2.07 ± 1.77	0.31
**Medicines**				
Metformin	0 (0%)	2 (3%)	20 (74%)	<0.0001
Gliclazide	0 (0%)	0 (0%)	3 (11.1%)	0.016
Empagliflozin	0 (0%)	0 (0%)	4 (14.8%)	0.012
Sitagliptin	0 (0%)	0 (0%)	2 (7.4%)	0.14
Liraglutide	0 (0%)	0 (0%)	2 (7.4%)	0.14
Statin	0 (0%)	7 (11.5%)	10 (37.0%)	0.007
ACE-i/ARB	0 (0%)	15 (24.5%)	15 (55.5%)	0.0015
Aspirin	0 (0%)	3 (5%)	2 (7.4%)	0.78
**Co-morbidities**				
Hypertension	4 (44.4%)	16 (26.2%)	19 (70.4%)	0.046
Dyslipidaemia	0 (0%)	6 (9.8%)	10 (37.0%)	0.002
Ischaemic heart disease	1 (11.1%)	1 (1.6%)	1 (3.7%)	0.16
MASLD/MASH	0 (0%)	3 (4.9%)	8 (29.6%)	0.003

Statistical analysis calculated using Kruskal–Wallis or Fisher's exact test. T2DM: Type 2 Diabetes Mellitus; BMI: body mass index; HbA1C: glycated haemoglobin; ACE-i: angiotensin-converting enzyme inhibitor; ARB: angiotensin receptor blocker; MASLD: Metabolic dysfunction-associated steatotic liver disease; MASH: Metabolic dysfunction-associated steatohepatitis.

**Table 2:** Clinical characteristics and metabolic profiling of non-MASLD, MASLD and MASH patients.

	Non-MASLD (n = 24)	MASLD (n = 14)	MASH (n = 3)	p-value
**Age**	49.7 ± 13.5	45.08 ± 13.1	56.33 ± 7.5	0.37
**Sex** (F)	19	10	3	0.72
**Ethnicity**				0.043
Asian/British Asian	2	0	1	
Black/black Caribbean	6	1	0	
White	14	8	1	
Other	0	4	1	
Mixed	2	1	0	
**BMI** (m^2^/kg)	45.89 ± 6.7	46.68 ± 11.7	46.68 ± 11.7	0.96
**HbA1C** (mmol/moll)	40.33 ± 6.1	42.36 ± 12.11	57.33 ± 24.21	0.24
**Cholesterol** (mg/dL)	4.5 ± 1.3	4.8 ± 0.99	4.57 ± 1.09	0.73
**Triglyceride** (mg/dL)	1.2 ± 0.68	1.8 ± 2.4	2.07 ± 1.77	0.31
**Medicines**				
Metformin	3 (12.5%)	4 (29%)	1 (33%)	0.36
Gliclazide	0 (0%)	1 (7%)	1 (33%)	0.05
Empagliflozin	0 (0%)	1 (7%)	1 (33%)	0.05
Sitagliptin	0 (0%)	0 (0%)	0 (0%)	
Liraglutide	0 (0%)	1 (7%)	0 (0%)	0.40
Statin	5 (36%)	3 (21.4%)	1 (33%)	0.86
ACE-i/ARB	11 (46%)	4 (28%)	2 (66%)	0.45
Aspirin	2 (8%)	1 (7%)	0 (0%)	0.99
**Co-morbidities**				
Hypertension	11 (46%)	7 (50%)	2 (66%)	0.89
Dyslipidaemia	3 (12.5%)	3 (21.4%)	1 (33%)	0.53
Ischaemic heart disease	1 (4.2%)	0 (0%)	0 (0%)	0.99
MASLD/MASH	0 (0%)	14 (100%)	3 (100%)	<0.0001

Statistical significance calculated using Kruskal–Wallis test or Fisher's exact test. Non-MASLD: non-Metabolic dysfunction-associated steatotic liver disease; MASLD: Metabolic dysfunction-associated steatotic liver disease; MASH: Metabolic dysfunction-associated steatohepatitis; BMI: body mass index; HbA1C: glycated haemoglobin; ACE-i: angiotensin-converting enzyme inhibitor; ARB: angiotensin receptor blocker.

Pearson's correlation coefficient for normally distributed data or Spearman's correlation coefficient for non-normally distributed data were used to evaluate the association between two continuous variables. For non-normally distributed categorical data used in Table 1, Table 2, Fisher's exact test and Kruskal–Wallis tests were used. Statistical significance was determined as p ≤ 0.05 after applying post-hoc tests.

#### Multivariate analysis

Multivariate analysis for WAT, plasma and liver oxylipins were performed using linear regression modelling after the dependant variable was normalised by log10 transformation of the data. Confounding factors including age, sex, ethnicity and fasting status were included in the model. Statistical significance was determined as p ≤ 0.05.

#### UPLC-MS/MS analysis

For the heatmap generation of WAT and plasma oxylipins, data were evaluated by applying the online metabolomics platform MetaboAnalyst 5.0.^66^ Briefly, normalized (log10 transformed) data was auto-scaled and clustered using Ward. The area under the curve (AUC) was calculated by using the univariate model in MetaboAnalyst. For oxylipin analysis in Table S3, FDR (Bejamini-Hochberg method) adjusted p-values have been included.

### Role of funders

The funding sources had no role in study design, data collection, data analyses, interpretation, or writing of the manuscript.

## Results

### Clinical characteristics and metabolic profile of the bariatric cohort

Eighty-eight patients undergoing bariatric surgery and nine lean patients were recruited to the study. Patients with obesity were split into those with a diagnosis of T2DM (N = 27) (as stated in clinical records or glycated haemoglobin ≥48 mmol/mol) and non-diabetic patients (N = 61; no clinical diagnosis of T2DM and glycated haemoglobin <48 mmol/mol) (Table 1). None of the lean patients were diabetic. Patients with obesity were more likely to have co-morbidities associated with metabolic syndrome including MASLD/MASH and dyslipidaemia (Table 1). Patients with T2DM were taking glucose-lowering drugs including Metformin (74%), Gliclazide (11.1%), Empagliflozin (14.8%), Sitagliptin (7.4%) and Liraglutide (7.4%) (Table 1).

### Adipocyte hypertrophy and hyperplasia in patients with obesity

**Fig. 1:**
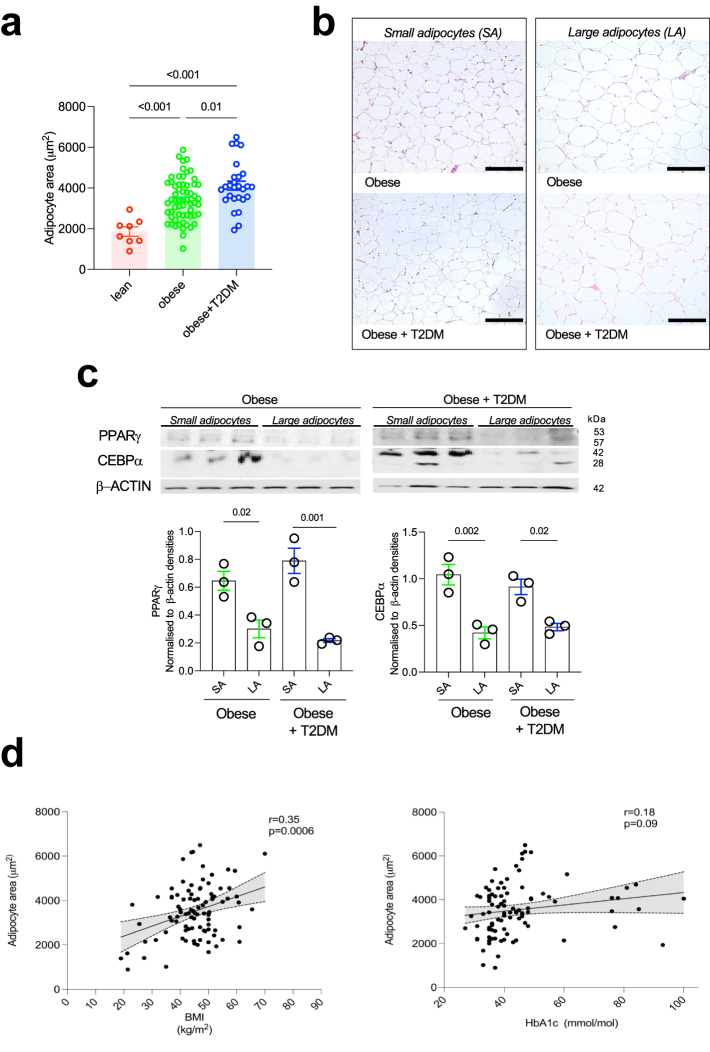
**Adipocyte hypertrophy and hyperplasia in patients with obesity**. (**a**) Average adipocyte area quantification in the WAT of lean patients (N = 9), patients with obesity (N = 61) and patients with obesity and T2DM (N = 27). (**b**) Representative H&E staining of omental WAT with presence of small adipocytes and large adipocytes in patients with obesity with and without T2DM. (**c**) Western Blot of WAT PPARγ and CEBP⍺ in patients with obesity with and without T2DM stratified according to the adipocyte size (N = 3 per group). (**d**) Correlation between adipocyte area (μm^2^) and BMI (kg/m^2^; left panel) and adipocyte area (μm^2^) and plasma glycated haemoglobin [HbA1c (mmol/mol); right panel]. Statistical significance was calculated using ANOVA with Tukey's multiple comparison test for (**a**) and (**c**) and using Pearson correlation coefficient for (**d**). Error bars represent SEM. Scale bars; 250 microns.

Uncontrolled hypertrophic expansion of WAT is linked with metabolic dysfunction while hyperplasia-mediated adipocyte expansion is considered to counteract the metabolic decline caused by hypertrophy.^10^^,^^67^^,^^68^ As such, adipose hyperplasia and hypertrophy are adaptive and non-adaptive biomarkers of obesity-induced WAT remodelling, respectively. We thus sought to study WAT hypertrophy and hyperplasia by measuring adipocyte size (hypertrophy) and whole tissue protein levels of PPARγ and CEBPA as markers of hyperplasia. As expected, lean controls had significantly smaller average adipocyte area than the cohort with obesity, with or without T2DM (Fig. 1a). Patients with obesity and T2DM had significantly larger adipocytes when compared to patients with obesity without T2DM (Fig. 1a and b). In order to measure hyperplasia, we focused on two transcription factors extensively studied during adipogenesis. PPARγ has been shown to be required for terminal adipocyte differentiation while CEBPα is crucial for WAT regeneration and expansion.^69^ Independently of T2DM status, there were significantly more PPARγ and CEBPα proteins expressed by small adipocytes when compared to large adipocytes in the WAT (Fig. 1c). BMI significantly correlated with mean adipocyte area, while glycated haemoglobin (HbA1c) did not correlate with average adipocyte size (Fig. 1d). In summary, while adipocyte size can differentiate the obese from lean, neither adipocyte hypertrophy nor hyperplasia seem to be robust markers of T2DM.

### WAT inflammation and collagen deposition during T2DM in patients with obesity

**Fig. 2:**
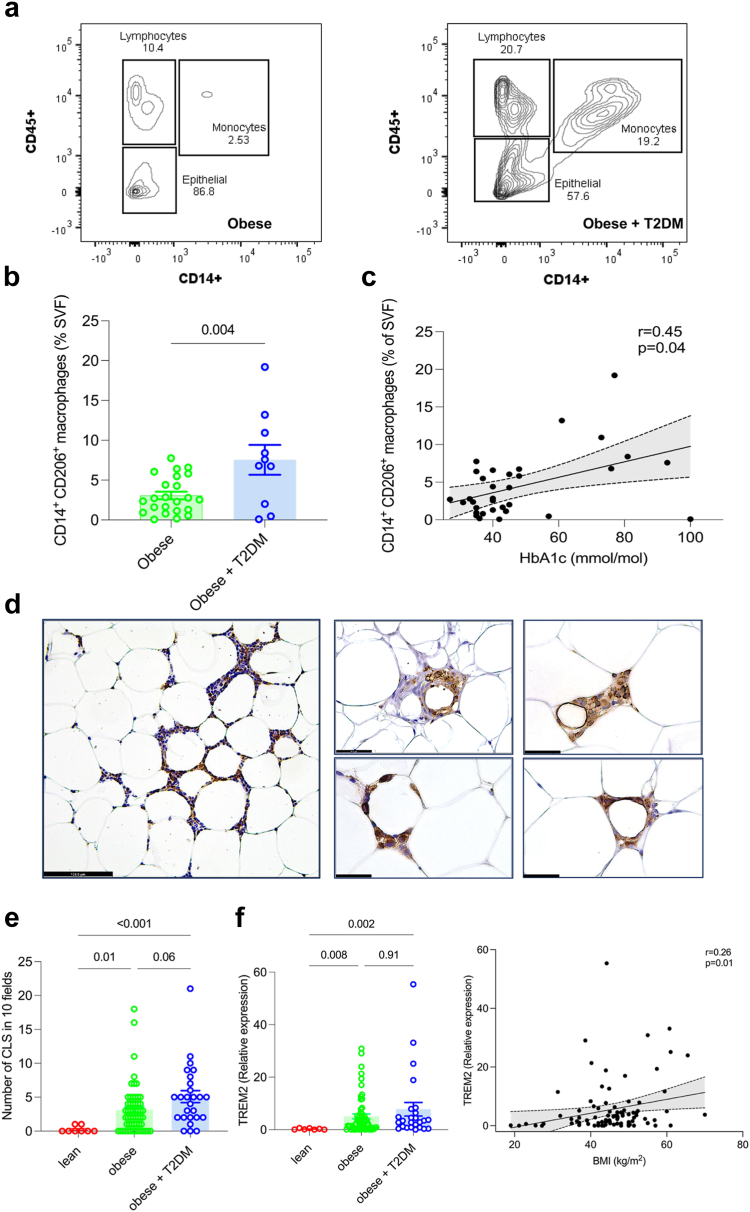
**WAT inflammation during T2DM in patients with obesity.** (**a**) Representative flow cytometry showing the increased proportion of monocytes/macrophages (CD45^+^, CD14^+^) in the SVF isolated from WAT of a patient with obesity and T2DM in comparison to a patient with obesity without T2DM. (**b**) Frequencies (%) of CD14^+^ CD206^+^ cells in the SVF of patients with obesity (N = 25) and patients with obesity and T2DM (N = 10). (**c**) Correlation between CD14^+^ CD206^+^cell frequencies and HbAc1 (mmol/mol) of patients with obesity (N = 33). (**d**) Representative IHC images for pan-macrophage marker CD68^+^ crown-like-structures (CLS) in the WAT of patients with obesity (N = 5 patients). (**e**) Quantification of CLS in the WAT of lean (N = 9), obese (N = 56) and patients with obesity and T2DM (N = 26). (**f**) *TREM2* mRNA expression (left panel) in the WAT of lean patients (N = 7), obese (N = 60) and patients with obesity and T2DM (N = 25). Correlation (right panel) between *CD68* and *TREM2* mRNA expression in the WAT of lean patients (N = 7), patients with obesity (N = 60) and patients with obesity and T2DM (N = 25). Error bars represent mean ± SEM. Statistical significance was calculated using Pearson's Correlation (**c**) and Spearman's correlation coefficient (**f**, right panel), Kruskal–Wallis test with Dunn's multiple comparison (**e**) and (**f**) Student's two tailed T-test (**b**); scale bars; 124.5 microns.

In order to identify WAT phenotypes that may correlate with T2DM, we next investigated tissue inflammation and collagen deposition. Monocytes and macrophages have been shown to be key players in chronic low-grade inflammation during obesity and their increasing number correlates with insulin resistance in the WAT.^3^ There were significantly more CD14^+^/CD45^+^ cells in T2DM patients’ SVF when compared to non-diabetic patients, with CD14+CD206+ monocyte/macrophage fraction significantly correlating with glycated haemoglobin (Fig. 2a, b and c, Figure S2). In keeping with the larger number of monocyte/macrophages in T2DM patients with obesity, we found a significant increase in the number of crown-like structures (CLS) in this group when compared to lean patients (Fig. 2d and e). In addition, *TREM2*, a marker of lipid-associated macrophages within the CLS of the WAT, showed a positive correlation between its mRNA levels and BMI (Fig. 2f).

**Fig. 3:**
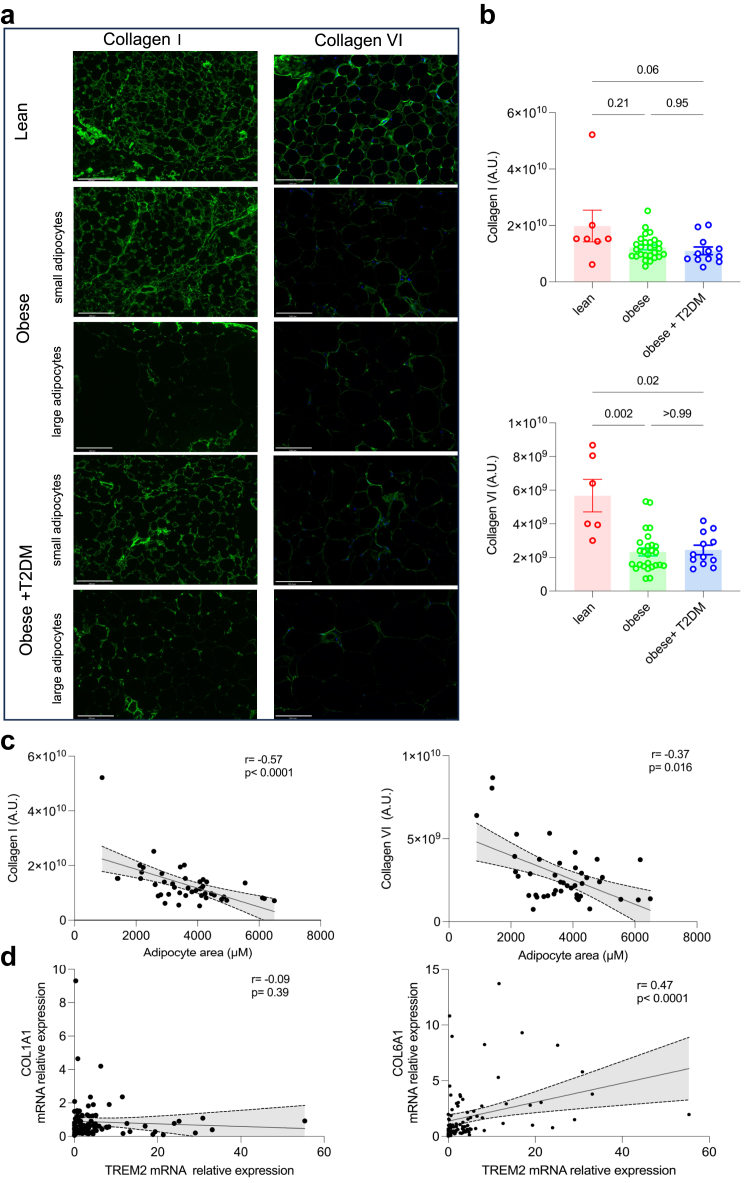
**WAT ECM remodelling during T2DM in patients with obesity**. (**a**) Representative immunofluorescence images for type I and type VI Collagen in lean patients or patients with obesity, with or without T2DM, with presence of small adipocytes or large adipocytes. (**b**) Type I collagen fluorescence (top panel) raw intensities (A.U.) in lean (N = 7), patients with obesity (N = 27) and patients with obesity and T2DM (N = 12). Type VI collagen fluorescence (bottom panel) raw intensities (A.U.) in lean (N = 7), obese (N = 27) and patients with obesity and T2DM (N = 12). (**c**) Correlation between type I Collagen fluorescence intensities (A.U.) and adipocyte area (μm^2^) (N = 46, left panel). Correlation between type VI Collagen fluorescence raw intensities (A.U.) and adipocyte area (μm^2^) (N = 46, right panel). (**d**) Correlation between *COL1A1* and *TREM2* mRNA levels (N = 91, left panel). Correlation between *COL6A1* and *TREM2* mRNA levels (N = 91, right panel). Statistical significance was calculated using Spearman's correlation coefficient (**c**) and (**d**) and Kruskal–Wallis test followed by Dunn's multiple comparison (**b**); error bars are mean ± SEM, scale bar 124.5 microns.

Several studies have shown that ECM homeostasis is a feature of WAT function, closely linked to adipocyte size and the inflammatory state of the WAT.^18^^,^^70^ Immunofluorescence and correlation analyses show collagen I having an overall reduction with increasing adipocyte size, while collagen VI shows heterogenous deposition with a difference in staining between lean individuals and patients with obesity (Fig. 3a, b and c). While collagen I showed no association with T2DM status (Fig. 3b) nor with macrophage markers (Fig. 3d, Figure S3) *COL6A1* transcript levels positively correlated with *TREM2* and *CD68* mRNA levels in the WAT (Fig. 3d and Figure S3). These results suggest that collagen I and VI associate with the structural integrity and inflammation of the WAT, respectively.

### WAT and plasma oxylipin quantification during obesity

**Fig. 4:**
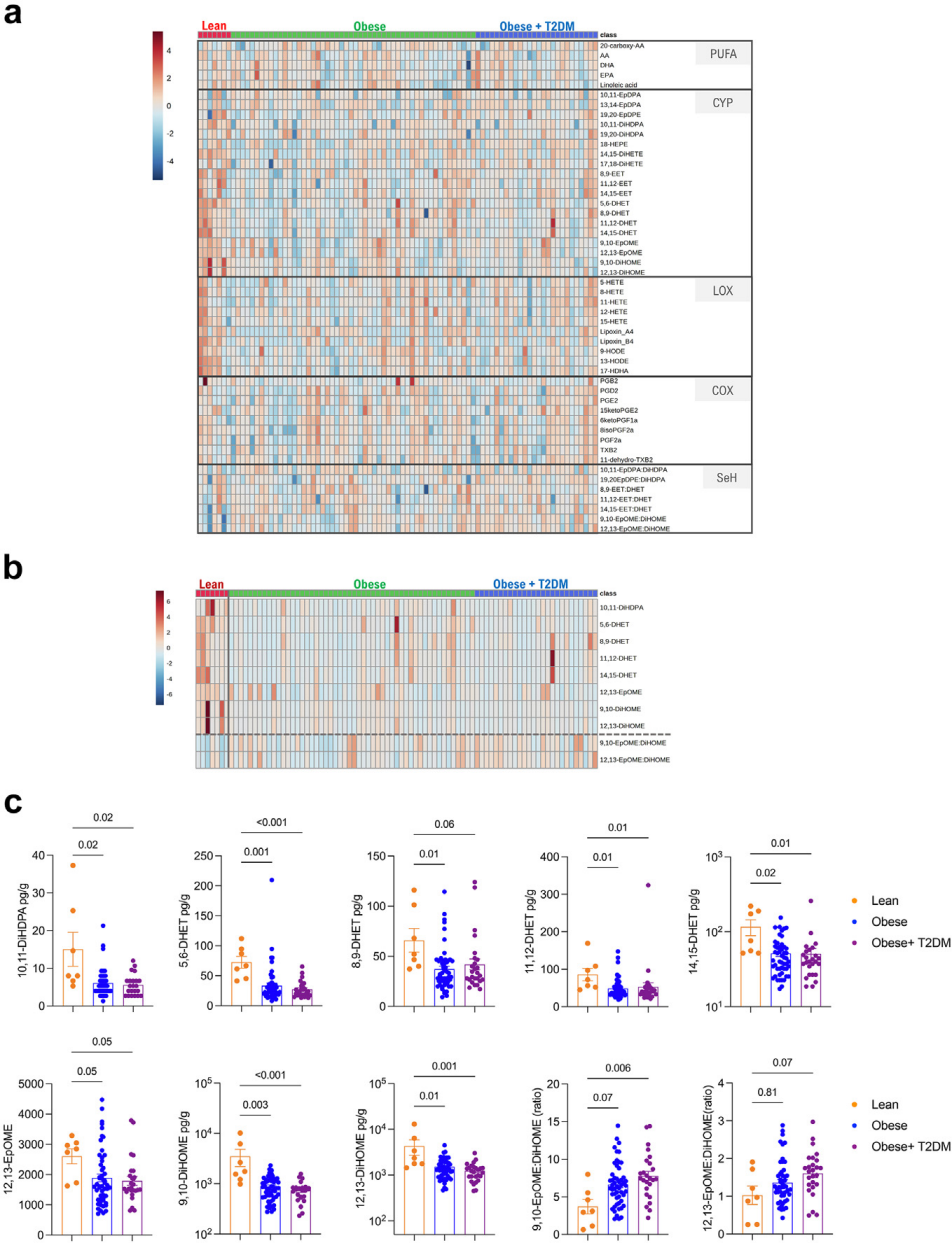
**WAT oxylipin quantification**. (**a**) Heatmap showing PUFAs, CYP-, LOX-, COX-derived oxylipins, and sEH activity readout in lean patients (N = 7), patients with obesity (N = 52) and patients with obesity and T2DM (N = 26). (**b**) 10 most significantly changing oxylipins (FDR <0.2). and sEH activity ratios (separated by a dashed line) determined by Kruskal–Wallis test (see also Table S3). (**c**) CYP-derived epoxides and diols in lean pateints (N = 7), patients with obesity (N = 52) and patients with obesity and T2DM (N = 26). Heatmaps (**a** and **b**) were generated on normalized data (log10 transformed) and clustered using Ward. Significance was tested by Kruskal–Wallis test followed by Dunn's multiple comparison (**c**).

**Fig. 5:**
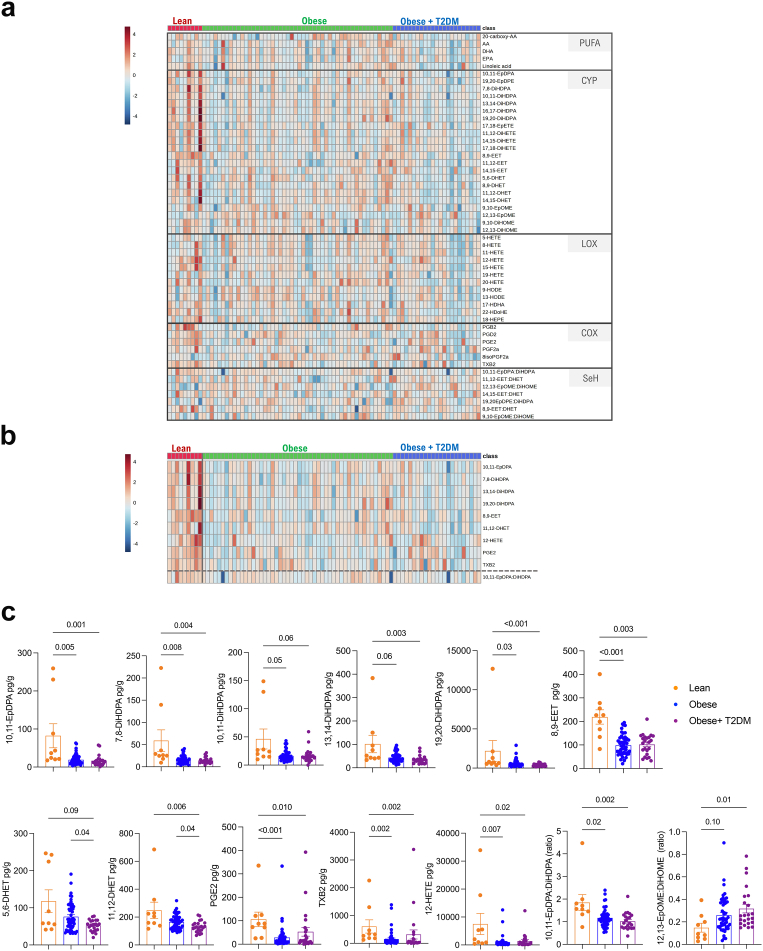
**Plasma oxylipin quantification**. (**a**) Heatmap showing PUFAs, CYP-, LOX-, COX-derived oxylipins, and sEH activity readout in lean patients (N = 9), patients with obesity (N = 50) and patients with obesity and T2DM (N = 25). (**b**) 10 most significantly changing (FDR<0.05) and sEH activity ratios (separated by a dashed line) determined by Kruskal–Wallis test. (**c**) LOX- , CYP- and COX-derived oxylipins in lean patients (N = 9), patients with obesity (N = 50) and patients with obesity and T2DM (N = 25). Heatmaps (**a** and **b**) were generated on normalized data (log10 transformed) and clustered using Ward. Significance was tested by Kruskal–Wallis test followed by Dunn's multiple comparison (**c**).

Having measured WAT hypertrophy, hyperplasia, inflammation and collagen deposition, we next focussed on the oxylipin profiling of the WAT in the same cohort by UPLC-MS/MS. Using the patient-derived omental WAT, we measured 20-carboxy-AA, AA, LA, EPA and DHA levels, as well as 38 unesterified different PUFA-derived oxylipins amongst which there were 19 CYP-derived, 10 LOX-derived and 9 COX-derived metabolites, including 7 readout of sEH activity (epoxide:diol ratio; Fig. 4a) and reported their significant changes between lean, obese and diabetic obese groups (Table S3). Strikingly, the most significant changes were observed within the 8 CYP-derived metabolites decreasing with obesity (10,11-DiHDPA, 5,6-DHET, 8,9-DHET, 11,12-DHET, 14,15-DHET, 12,13-EpOME, 9,10-DiHOME, and 12,13-DiHOME), especially in AA, LA and DHA-derived fatty acid diols (Fig. 4b and c). 9,10-EpOME:DiHOME and 12,13-EpOME:DiHOME ratios were increased in patients with obesity, indicative of reduced sEH activity (Fig. 4b and c). We next performed similar analyses using the patient-derived plasma (Fig. 5a). The most significant changes were observed in mostly CYP-derived oxylipins (including many DHA-derived) and down-regulated in patients with obesity when compared to lean individuals (Fig. 5b). Notably, the commonly down-regulated oxylipins in obese WAT and plasma included 3 CYP-derived diols (5,6-DHET, 10,11-DiHDPA 11,12-DHET) (Fig. 5b and c). Consistent with the decreased sEH activity in WAT, 12,13-EpOME:DiHOME was similarly increased in the plasma of patients with obesity when compared to lean individuals (Fig. 5b and c). These results suggested a dysregulation of the CYP pathway, which can result from changes in the expression levels of CYP and/or sEH enzymes (*EPHX1* and *EPHX2*). No significant changes were found in the expression of *CYP2J2*, *CYP2S1*, *CYP2C8*, *CYP2C9*, *EPHX1*, *EPHX2* between lean, obese and diabetic obese groups (Figure S4). The results were similar for major LOX enzymes (*ALOX5*, *ALOX12* and *ALOX15*; Figure S4).

### Inflamed WAT associates with increased 12,13-EpOME:DiHOME

**Fig. 6:**
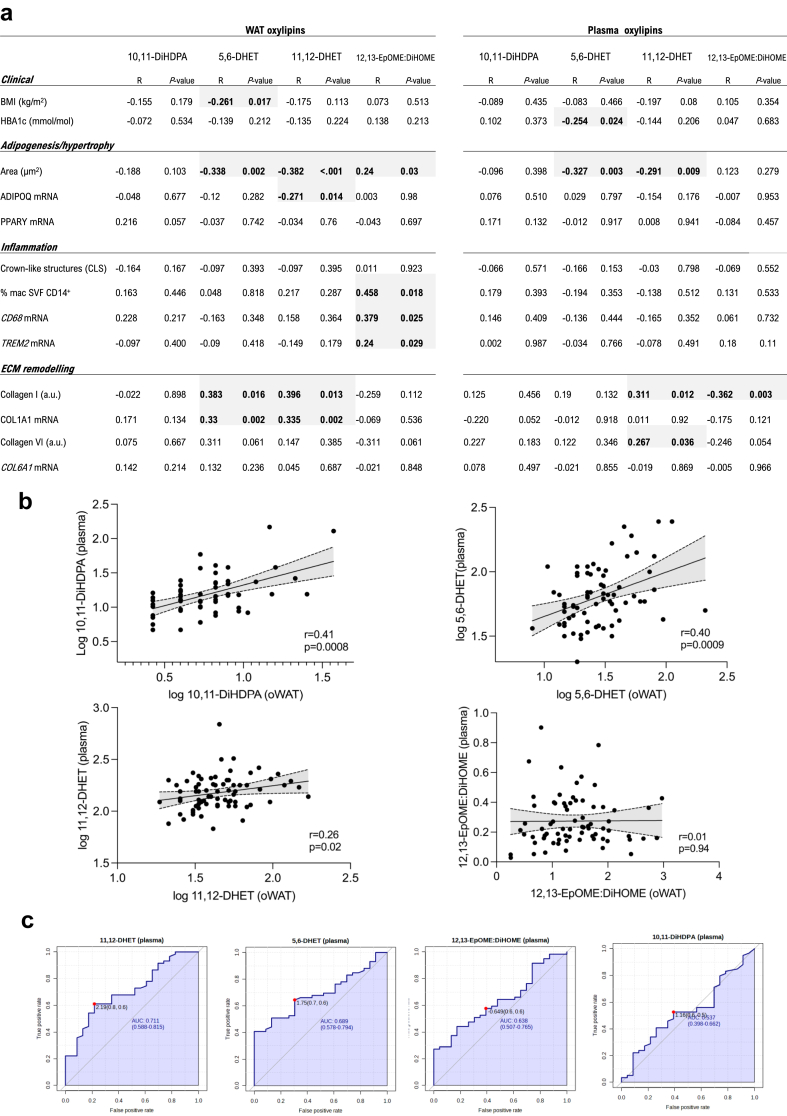
**Integration of WAT and plasma oxylipins with WAT phenotypes**. (**a**) Correlation of WAT (N = 83; left) and plasma (N = 80; right) 10,11-DiHDPA, 5,6-DHET, 11,12-DHET and 12,13-EpOME:DiHOME with clinical (BMI, HbA1C) and WAT (adipogenesis/hypertrophy, inflammation, ECM remodelling) phenotypes. Grey highlights denote significant associations. (**b**) Correlation of the 3 CYP-derived epoxides (pg/g) and 12,13-EpOME:DiHOME ratio between plasma and WAT (N = 72). (**c**) AUC curves of plasma levels of 3 CYP-derived epoxides (pg/g) and 12,13-EpOME:DiHOME ratio for T2DM diagnostic accuracy (N = 82). Significance was tested by Spearman's correlation coefficient (**a** and **b**).

The commonly down-regulated CYP-derived diols and up-regulated 12,13-EpOME:DiHOME in obese WAT and plasma were further analysed in relation to (i) their association with WAT phenotypes (Fig. 1, Fig. 2, Fig. 3), (ii) their cross-tissue correlation (i.e. WAT vs. plasma), (iii) their relative predictive value of T2DM. Before doing so, we verified whether the level of these 3 fatty acid diols and 12,13-EpOME:DiHOME have cofounders such as age, ethnicity and sex in the bariatric cohort. Multivariate linear regression analysis showed that when metabolite measurements were adjusted for cofounders, 5,6-DHET, 10,11-DiHDPA and 12,13-EpOME:DiHOME were significantly altered in obese WAT (Figure S5). Similarly, the adjusted plasma metabolites showed that 10,11-DiHDPA and 12,13-EpOME:DiHOME were also significantly modulated in obesity (Figure S5). Following these observations, we investigated whether this obesity-associated oxylipin signature is associated with WAT dysfunction, and whether it could be a biomarker of pathological changes occurring in the adipose tissue. Clinical data (BMI, HbA1c) and phenotypic markers of adipose tissue (adipogenesis/hypertrophy, inflammation, collagen deposition) were correlated with the 3 CYP-derived metabolites and 12,13-EpOME:DiHOME (Fig. 6a). Whether measured in plasma or WAT, fatty acid diols such as 5,6-DHET and 11,12-DHET significantly decreased as the size of adipocytes increased (Fig. 6a). 11,12-DHET in WAT and plasma significantly increased with adipose tissue collagen I (Fig. 6a), which associates with adipocyte size (Fig. 3c). On the other hand, 12,13-EpOME:DiHOME associates significantly with WAT inflammation (% of macrophages in SVF, CD68 and TREM2 mRNA levels). The 3 diols measured in the WAT of the cohort correlate significantly with plasma measurements, though 12,13-EpOME:DiHOME did not show any correlation (Fig. 6b). The area under the curve (AUC) was then used to compare the T2DM diagnostic accuracy of 10,11-DiHDPA, 5,6-DHET, 11,12-DHET and 12,13-EpOME:DiHOME (Fig. 6c). Finally, another cofounding factor is the wide-usage of metformin in our bariatric cohort patients. Indeed, 74% of T2DM patients with obesity were prescribed metformin (Table 1). In order to test whether metformin can have an effect on WAT-derived oxylipin production, we cultured patient-derived omental fat explants with metformin *ex vivo* and performed UPLC-MS/MS afterwards. We first confirmed that a clinically relevant concentration of metformin does not affect the viability of the WAT cells (Figure S6a and b). It was shown that serum levels of metformin peak at 20 μM^71^ and that WAT does not have high uptake of metformin,^72^ suggesting that the *in vivo* effect of metformin is likely to be in micromolar range in humans. We thus incubated 7 WAT explants from patients with obesity with metformin (1 μM, 48 h) before performing UPLC-MS/MS. With the exception of 5,6-DHET, there was no significant change in quantities of 10,11-DiHDPA and 11,12-DHET and 12,13-EpOME:DiHOME measured in the explants (Figure S6).

In summary, after a data filtering approach that included (i) conserved association between WAT and plasma, (ii) significance retained after multivariate linear regression, (iii) metformin-independent association, the results show the association of 12,13-EpOME:DiHOME with obesity. Specifically, increased 12,13-EpOME:DiHOME associate with WAT inflammation (macrophage infiltration markers). Importantly, these results suggest reduced LA-derived tissue activity of sEH (12,13-EpOME:DiHOME) with worsening WAT homeostasis.

### Increased hepatic epoxide:diol is conserved during MASLD

**Fig. 7:**
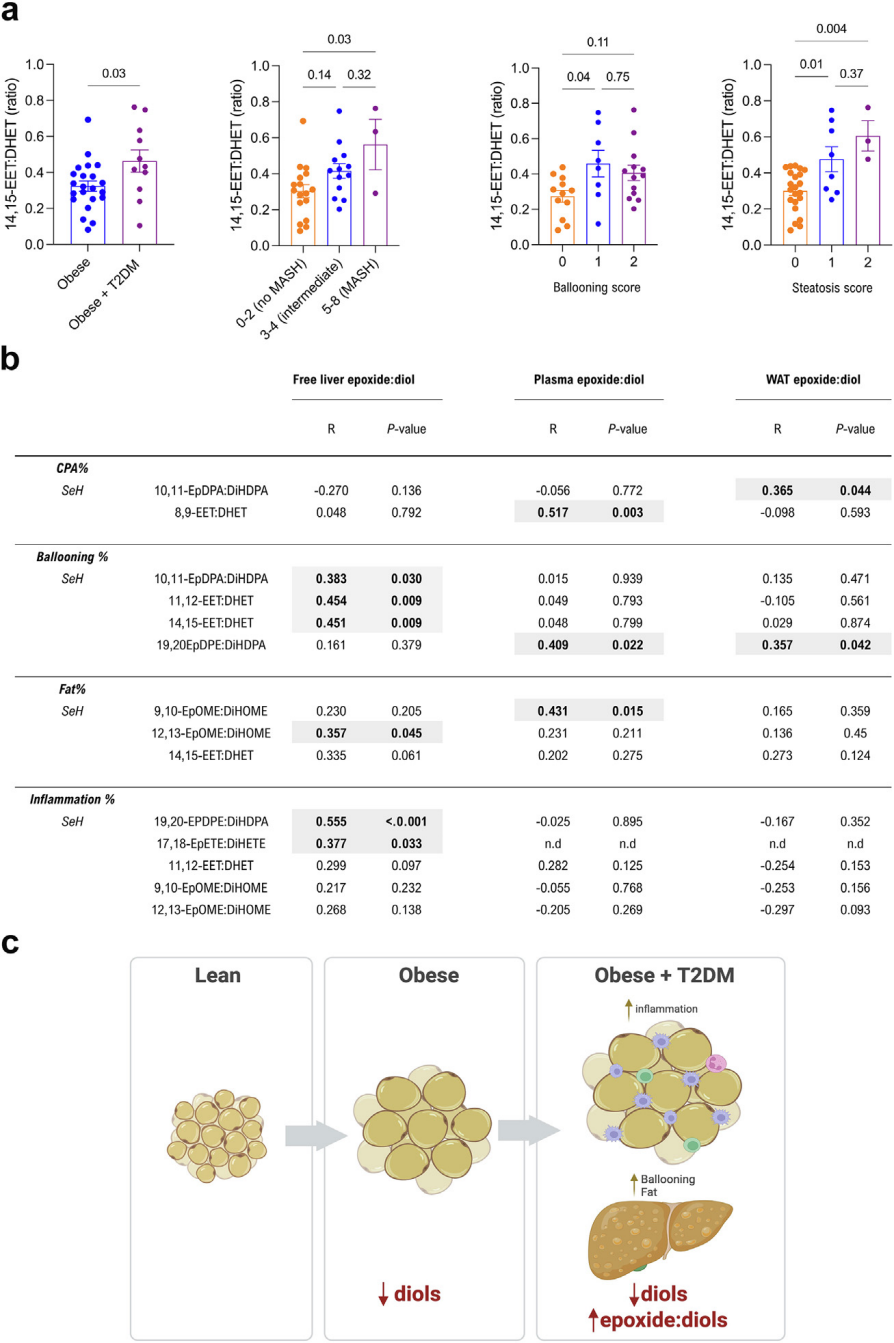
**Increased hepatic epoxide:diol is conserved during MASLD**. (**a**) sEH surrogate 14,15-EET:DHET in liver biopsies stratified according to patients with obesity (N = 22) and patients with obesity and T2DM (N = 11) or MASH [NAS scoring, 0–2 no MASLD (N = 17), 3–4 intermediate/MASLD/MASH (N = 13), 5+ MASH (N = 3)] or ballooning score (0–2; score 0: N = 12, score 1: N = 8, score 2: N = 13) or steatosis score (0–3; score 0: N = 22, score 1: N = 8, score 2: N = 3, score 3: N = 0). (**b**) Correlation of sEH surrogates, measured in each tissue (liver, plasma, WAT) by UPLC-MS/MS with markers of MASLD, measured by machine learning (**See Methods**; CPA%, ballooning%, fat%, inflammation %) for each liver biopsy (N = 35). Grey highlights denote significant associations. (**c**) Schematic summary of CYP-sEH pathway evolution between lean and obese states in the WAT and during WAT inflammation and hepatic steatosis. Significance was tested by student's t-test (2-sided) (**a**), Kruskal–Wallis followed by Dunn's multiple comparison (**a**) and Spearman's correlation coefficient (**b**).

An unhealthy WAT is often indicative of changes in the liver lipid content and progression toward MASLD. As dysfunctional WAT associates with a specific oxylipin signature, we next sought to investigate the liver oxylipin profiling within our bariatric cohort. A subgroup of 41 patients from our patients with obesity undergoing bariatric surgery (Table 2) were sampled for liver biopsies, and PUFA/oxylipin profiling (20-carboxy-AA, AA, LA, EPA and DHA levels and 48 PUFA-derived oxylipins) by UPLC-MS/MS. Liver biopsies were scored for existence of MASH, steatosis, ballooning, inflammation and collagen deposition (see **Methods**). Consistent with the decreased sEH activity found in the inflamed WAT, hepatic 14,15-EET:DHET was significantly increased in obesity with T2DM, independently of the sex, age and ethnicity (Fig. 7a, Figure S7a). This increase was not linked to changes in hepatic mRNA levels of *EPHX1* and *EPHX2* as both microsomal and soluble isoforms' expression were not significantly altered with T2DM in patients with obesity (Figure S8). 14,15-EET:DHET also increased significantly with some MASLD clinical phenotypes (Fig. 7a). This association does not hold when 14,15-EET:DHET is calculated based on total amounts (free and esterified) of 14,15-EET and 14,15-DHET (Figure S7b and c). The positive association of free 14,15-EET:DHET with T2DM let us investigate all measured readout of free sEH activities in relation to the severity of MASLD (Fig. 7b). The correlation of different epoxide:diol with % collagen proportionate area (CPA), ballooning, fat and inflammation, revealed significant positive associations (Fig. 7b). Multivariate analysis retained the significance for those related to % ballooning and fat (10,11-EpDPA:DiHDPA, 11,12-EET:DHET, 14,15:EET:DHET, 12,13-EpOME:DiHOME; Figure S9). The association of 19,20-EpDPE:DiHDPA and 17,18-EpETE:DiHETE with hepatic inflammation was lost after multivariate linear regression (Figure S9). When analysed according to the liver pathology criteria (ballooning and steatosis), several epoxide:diol ratios were also increased in plasma and WAT (Fig. 7b). In summary, the increased epoxide:diol observed in inflamed WAT is conserved during the ballooning and steatosis observed in MASLD livers. While hepatic 14,15-EET:DHET increases with T2DM, 12,13-EpOME:DiHOME, a marker of WAT inflammation (Fig. 6a), is also increased with steatosis in the liver. Overall these results suggest that while obesity is associated with overall reduced fatty acid diols, obesity-associated WAT inflammation and the severity of MASLD are associated with dampened sEH activity (Fig. 7c).

## Discussion

During obesity, the early interplay between a ‘fat-buffering’ endocrine WAT and liver homeostasis can be defective and cause the chronic metabolic complications that may occur at later stages. Adipokines and lipokines are well-established mediators of the WAT-liver crosstalk^73^^,^^74^ and among those, PUFA-derived oxylipin superfamily exerts pleiotropic functions on organ homeostasis. However, little is known about their tissue-specific evolution during metabolic syndrome.

Our omental fat phenotyping in the bariatric cohort confirms well-described features of obese WAT and makes a distinction between readout of inflammation/fibrosis and hypertrophy/hyperplasia. A robust indicator of WAT inflammation is the presence of mononuclear phagocytes^75^, ^76^, ^77^ and we describe increasing numbers of macrophage and CLS numbers with T2DM. On the other hand, we report that adipocyte hypertrophy is associated with down-regulation of PPARγ and CEBPα, independently of the occurrence of T2DM. We found that type I collagen inversely correlated with average adipocyte size, independently of T2DM status and WAT macrophage abundance. Conversely, type VI collagen positively correlated with WAT macrophage infiltration despite being reduced in patients with obesity. Previous studies corroborated these findings, demonstrating that increased collagen VI deposition is associated with worsening metabolic function and WAT inflammation during chronic obesity.^78^, ^79^, ^80^ Importantly, macrophage depletion in WAT explants isolated from patients undergoing bariatric surgery led to reduced type VI collagen and improved insulin sensitivity *ex vivo*.^81^ In summary, our WAT phenotyping shows that adipocyte hypertrophy (i.e. adipocyte size), type I collagen, PPARγ/CEBPα reduction (hyperplasia) are indicative of homeostatic remodelling during obesity, while macrophage numbers, type VI collagen are cellular and histological markers of progression toward T2DM.

Oxylipins are oxygenated bioactive lipid mediators with intracellular and extracellular (via G protein-coupled receptors) signalling actions in tissues. Depending on the type of PUFA they are derived from and the type of primary oxidative enzyme, they can have pro-inflammatory or pro-resolving properties that are also reported in the adipose tissue and liver.^21^^,^^82^, ^83^, ^84^ In this study, we identify significant changes in the CYP epoxide/diol pathway in the WAT, liver and plasma of individuals with metabolic syndrome. Our results show CYP-derived diols, whether AA, LA or DHA-derived, being prominently reduced in the WAT and plasma of individuals with obesity when compared to lean ones. Notably, a systemically conserved marker of obesity, 12,13-EpOME:DiHOME, increases with WAT inflammation (macrophage infiltration), suggestive of decreased sEH activity with progressively dysfunctional WAT. The epoxide/diol profiles in the liver biopsied from patients with obesity corroborated these findings, suggesting an overall reduced sEH activity (increased 14,15-EET:DHET) when patients are stratified according to the occurrence of T2DM. More generally, different epoxide:diol ratios (10,11-EpDPE:DiHDPA, 11,12-EET:DHET, 14,15-EET:DHET) correlated positively with ballooning and steatosis of the obese liver. Interestingly, 12,13-EpOME:DiHOME, was also associated with hepatic steatosis, suggesting that is a common marker of WAT inflammation and liver steatosis during obesity. Overall, the results suggest that the fatty acid diol reduction may be associated broadly with obesity and reduced sEH activity (increased 12,13-EpOME:DiHOME) is indicative of metabolic dysfunction characterized by WAT inflammation and hepatic steatosis.

Here we show that a possibly dampened sEH activity and reduced fatty acid diol levels, characterize obesity and its metabolic complications including MASLD. While the literature on CYP-sEH pathway association with human metabolic syndrome refers to contradictory findings^39^^,^^40^^,^^42^, ^43^, ^44^, ^45^^,^^47^^,^^49^, ^50^, ^51^, ^52^, ^53^ (e.g. changes in hepatic epoxide levels were linked to either increased or decreased CYP epoxygenase activity, depending on the stage of disease and the type of patients in MASLD/MASH),^39^^,^^49^ there is converging evidence suggesting that the metabolic component of obesity is characterized by reduced diols and/or sEH activity. Firstly, lower proportions of CYP-derived diols negatively correlate with HOMA2-IR in the subcutaneous WAT of humans.^43^ Secondly, elevated plasma total 14,15-DHET have been suggested to be protective during early phases of diabetes with an inverse association of 14,15-DHET with BMI.^50^ Plasma levels of another diol, 12,13-DiHOME, has been inversely corelated with BMI, insulin resistance and circulating markers of liver function,^85^ a finding replicated by other recent studies.^52^^,^^86^ Of note, an obesity-induced reduction in diols and/or sEH activity has also been observed in the WAT using animal models of obesity. In cafeteria diet fed rats and high-fat diet induced obesity in mice, a generalized down-regulation WAT CYP-derived epoxides and diols (including 12,13-DiHOME) confirm the findings presented here and the above-mentioned studies conducted in humans.^87^^,^^88^ Finally, sEH inhibition does not modify insulin sensitivity in patients with obesity and prediabetes^89^ and loss of EPHX1 activity causes lipoatrophic diabetes syndrome.^90^ Collectively, these data suggest that early metabolic complications of obesity are characterized by dysfunctional CYP-sEH pathway that manifests with reduced sEH activity and diols in WAT and liver. Further studies need to clarify the relative activities of sEH enzymes with respect to CYP epoxygenase activities taking place upstream.

Epoxides are commonly described in the literature as resolving/regenerative and anti-inflammatory mediators^91^, ^92^, ^93^ as opposed to pathogenic diols.^94^ As such, the *in situ* activity of sEH has been used as a proxy for pathogenicity and/or inflammation and inhibition of sEH activity has been associated with improved disease outcome, especially in pathologies involving the vascular endothelium in their etiology.^95^, ^96^, ^97^ Despite being effective in diabetic retinopathy,^94^ sEH inhibition does not modify insulin sensitivity^89^ suggesting that CYP-sEH pathway acts on different levels in T2DM.^98^ This is further supported by the multitude of pathways linked to sEH such as endothelial barrier function, ER stress, apoptosis and oxidative stress. In that sense, the improved glycaemia after sEH inhibition in animal models can be attributed to its action on pancreatic islets *in vivo*^99^, ^100^, ^101^ and to ER stress *in vitro*.^102^ In view of our results and those obtained by others,^43^^,^^50^^,^^52^^,^^85^, ^86^, ^87^, ^88^^,^^90^ therapies based on sEH inhibition in metabolic syndrome should consider a potentially defective CYP-sEH pathway in metabolic tissues such as WAT and the liver of patients with obesity. This scenario may be different during other complications of T2DM (retinopathy, nephropathy, cardiomyopathy) whereby an induction of the pathway (increased sEH activity) may occur in a tissue-and context-specific manner. Evidence that supports this hypothesis comes from the lack of association of EETs in diabetes despite a negative association with cardiovascular complications of diabetes.^50^^,^^103^ In addition to targeting sEH activity, another upcoming promising therapeutic approach is to target oxylipin receptors for modulating vascular function related to cardiometabolic disease.^104^

*What causes a defective CYP-sEH pathway during obesity?* The intracellular epoxide concentration reflects the balance between the production of catalysed CYP epoxygenases and hydration by the corresponding sEH downstream. Our results show that obesity is characterized by a generalized down-regulation of diols in WAT and plasma without any significant changes in the expression of *EPHX* enzyme isoforms, suggesting a reduced enzymatic activity of sEH. If obese WAT is characterized by a reduced sEH activity in patients, how can we then reconcile our results with the previously reported increased sEH expression during adipocyte differentiation^61^^,^^105^^,^^106^? The answer to this question may lie in the distinction made between ‘healthy obesity’ (active adipogenesis) and ‘unhealthy obesity (halted adipogenesis). In terms of healthy adipocyte differentiation, the current evidence points towards a pro-adipogenic role of diols as opposed to anti-adipogenic epoxides. Indeed, *Ephx1* deletion in pre-adipocytes abolished adipocyte differentiation^90^ while rat epoxygenase *Cyp2j4* deletion causes accelerated adipogenesis in mesenchymal stem cells, increased PPARγ in macrophages and wait gain in ageing rats.^87^^,^^107^ During unhealthy obesity, the hypertrophic adipocytes are characterized by reduced PPARγ and CEBPa (this report and^87^) which indicates an inhibition of the adipogenesis in the WAT of morbidly patients with obesity undergoing bariatric surgery. This may explain the plausible reduced sEH activity that we observe in metabolic organs such as WAT and the liver. The precise mechanistic link assuring the interdependency between the CYP-sEH pathway and adipogenesis in metabolic tissues remain to be identified. Nonetheless, strategies aiming to supplement LA-derived diols such as 12,13-DiHOME in obesity are sound and can remedy the defective adipocyte differentiation observed during obesity. This is in line with what was described for adiponectin which enhances adipocyte lipid storage and adipogenesis, preventing ectopic lipid accumulation.^108^ In general, a more comprehensive understanding of the role of fatty acid diols in adipose tissue, including their concentration-dependent actions,^95^ will be key in understanding their implication during obesity.

Our study presents some limitations. Lean control patients undergoing upper gastrointestinal surgery for local oesophageal or gastric carcinoma were used as controls. Although obtaining entirely healthy visceral WAT presents challenges, these lean (normal BMI) samples (see also Methods) showed small adipocytes and absence of prominent WAT inflammation, placing them on the healthy spectrum of WAT phenotypes. In terms of liver histology, the severity of MASLD in our cohort is mild with few patients presenting a definitive MASH. Hence, our findings may relate to early complications of obesity in the liver. Liver biopsies are taken from the cohort with obesity during bariatric surgery so the liver oxylipins were analysed in the context of evolving liver pathology; not between ‘lean’ and ‘obese’ states. Furthermore, oxylipins can be esterified to complex lipids, which can be quantified in plasma as the predominant form.^50^^,^^109^ In this study, we measured both forms in the liver and showed that the association of free hepatic 14,15-EET:DHET was lost with total 14,15-EET:DHET. This suggests that the systematic measurement of both pools in plasma and adipose tissue can refine the cross-tissue dynamics of these bioactive lipids. Finally, the sEH activity is inferred from epoxide:diol ratios, a surrogate enzymatic activity, which does not inform on which PUFA and CYP-derived epoxide are predominantly utilized by the tissue. The tissue PUFA composition may not reflect accurately the oxylipin content,^110^ so future studies should aim to characterize the relative abundance of PUFAs, epoxides and diols with regard to *ex vivo* measured sEH enzymatic activities in tissues.

In summary, these findings reveal a potentially defective CYP-sEH pathway, suggesting an overall reduced WAT and liver sEH activity during metabolic syndrome. Increased 12,13-EpOME:DiHOME can be considered as a marker of worsening metabolic syndrome in patients with obesity, which suggests fatty acid diol supplementation as an alternative therapeutic approach.

## Contributors

J.B. conceived and supervised the study. C.H., A.O., L.H., M.L.E., J.H.K., F.L., performed the experiments. C.H. recruited the patients and collected the samples with help from X.T., A.O. R.B. and J.B. Data collection, analysis and interpretation were done by C.H., J.B., and E.P. The machine learning-based liver histology was performed by R.F., P.M. and R.G. Bariatric biopsy collection was performed by L.H., S.P., K.M. Data were verified by C.H., J.B., M.L.E., D.C.Z. and E.P. The manuscript was written by J.B. and C.H. The manuscript was edited by J.B., C.H., M.T., M.L.E., D.C.Z., G.Z. All authors read and approved the final version of the manuscript.

## Data sharing statement

The individual-level data used in this study cannot be publicly shared due to ethical approval. Data set will be made available upon request through contact with the corresponding author. For sharing of data within a scientific collaboration any proposal should be directed to the corresponding author. Data will only be shared in accordance with legal frameworks, and when the integrity of the individual study participant can be guaranteed. This will be decided by the corresponding author on a case-by-case basis.

## Declaration of interests

The authors declare no conflict of interest.

## Appendix A Supplementary data

Supplementary Figure 9

Supplemental Western Blot File

Supplementary Table 3

Certicate of compliance Type VI collagen

Certificate of Analysis CD14

Certificate of Analysis CD206

Certificate of Analysis CD3

Certificate of Analysis CD45

Certificate of Analysis CD68

Certificate of Analysis CEBPA

Certificate of Analysis Collagen I

Certificate of Analysis PPARY
