# Supplementary material for: Multi-tissue profiling of oxylipins reveal a conserved up-regulation of epoxide:diol ratio that associates with white adipose tissue inflammation and liver steatosis in obesity
Source: eBioMedicine. 2024 Apr 26;103:105127. doi: 10.1016/j.ebiom.2024.105127 (PMC11061246; doi:10.1016/j.ebiom.2024.105127)
Supplement: Supplementary Figure 1 [file mmc1.pdf]

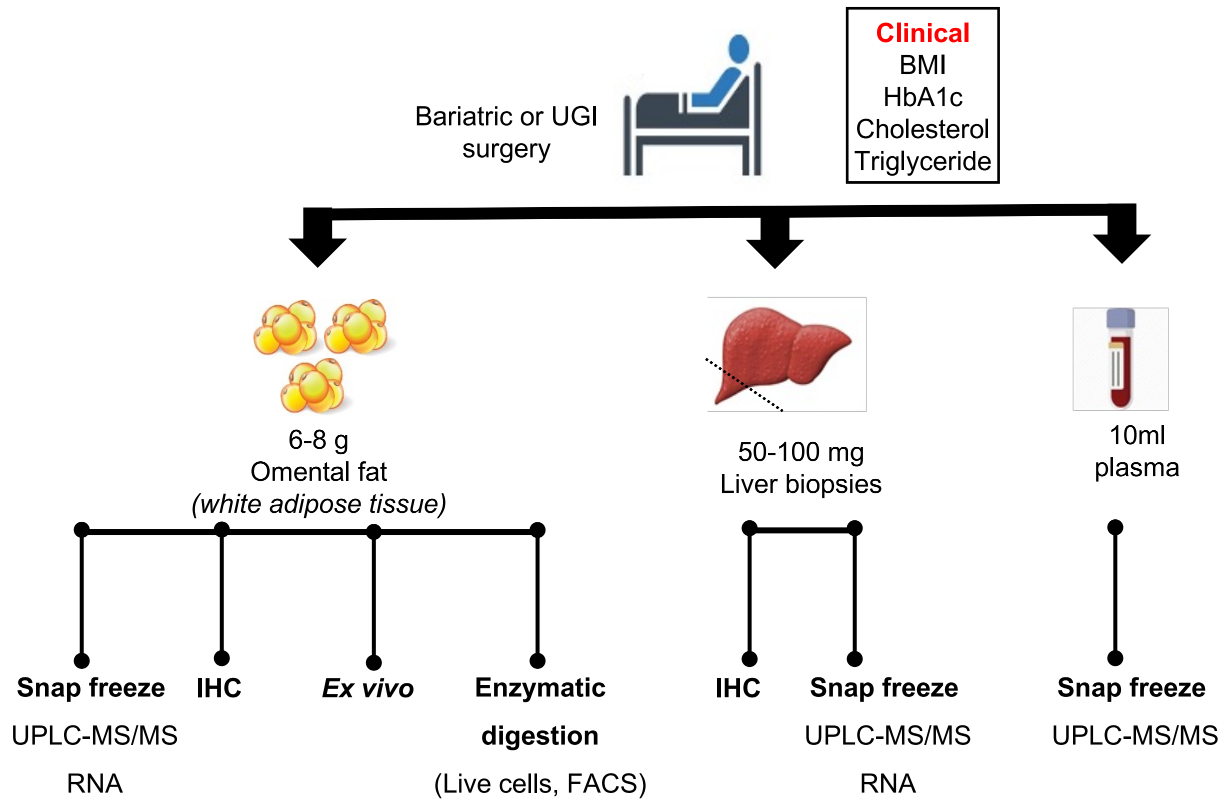

Supplementary Figure 1. Study Design and schematic flowchart of the bariatric and upper gastrointestinal (UGI) surgery sample collection. Liver biopsies were only taken in N=41 bariatric surgery patients. IHC, immunohistochemistry.
