# Supplementary material for: Multi-tissue profiling of oxylipins reveal a conserved up-regulation of epoxide:diol ratio that associates with white adipose tissue inflammation and liver steatosis in obesity
Source: eBioMedicine. 2024 Apr 26;103:105127. doi: 10.1016/j.ebiom.2024.105127 (PMC11061246; doi:10.1016/j.ebiom.2024.105127)
Supplement: Supplementary Figure 2 [file mmc2.pdf]

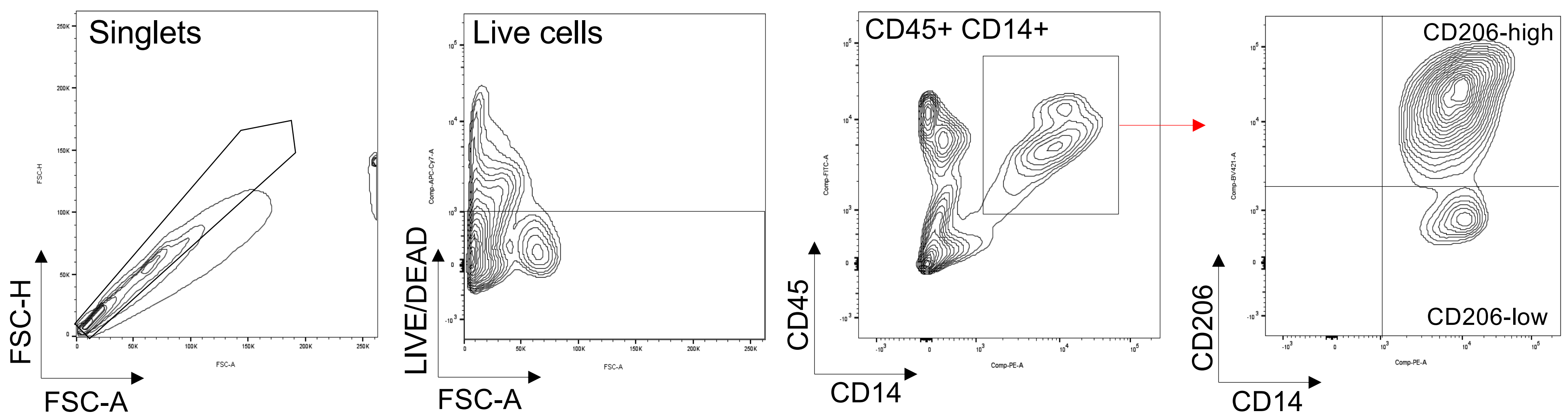

Supplementary Figure 2. Gating strategy for monocyte/macrophage markers in stromal vascular fraction (SVF) isolated from WAT of bariatric surgery patients; from left to right, the panels indicate (i) singlets, (ii) live cells, (iii) CD45+CD14+ cells, (iv) CD206+ cells. The red arrow indicates the direction of gating.
