# Supplementary material for: Multi-tissue profiling of oxylipins reveal a conserved up-regulation of epoxide:diol ratio that associates with white adipose tissue inflammation and liver steatosis in obesity
Source: eBioMedicine. 2024 Apr 26;103:105127. doi: 10.1016/j.ebiom.2024.105127 (PMC11061246; doi:10.1016/j.ebiom.2024.105127)
Supplement: Supplementary Figure 3 [file mmc3.pdf]

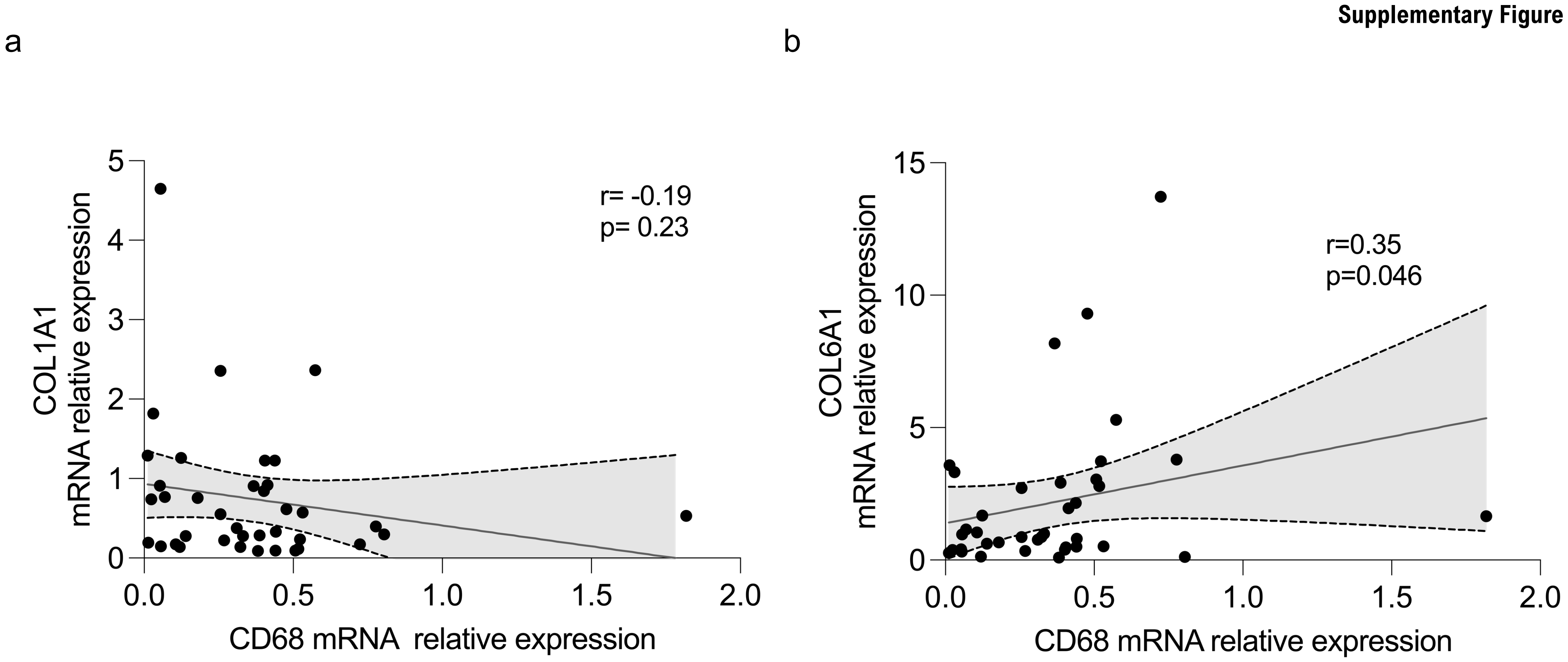

Supplementary Figure 3. **Macrophage infiltration and type I, type VI collagens in the WAT**. Correlation between COL1A1 (**a**) and COL6A1 (**b**) mRNA levels and CD68 mRNA levels. N=38; statistical significance was calculated using Spearman's correlation coefficient (A) and (B).
