# Supplementary material for: Multi-tissue profiling of oxylipins reveal a conserved up-regulation of epoxide:diol ratio that associates with white adipose tissue inflammation and liver steatosis in obesity
Source: eBioMedicine. 2024 Apr 26;103:105127. doi: 10.1016/j.ebiom.2024.105127 (PMC11061246; doi:10.1016/j.ebiom.2024.105127)
Supplement: Supplementary Figure 4 [file mmc4.pdf]

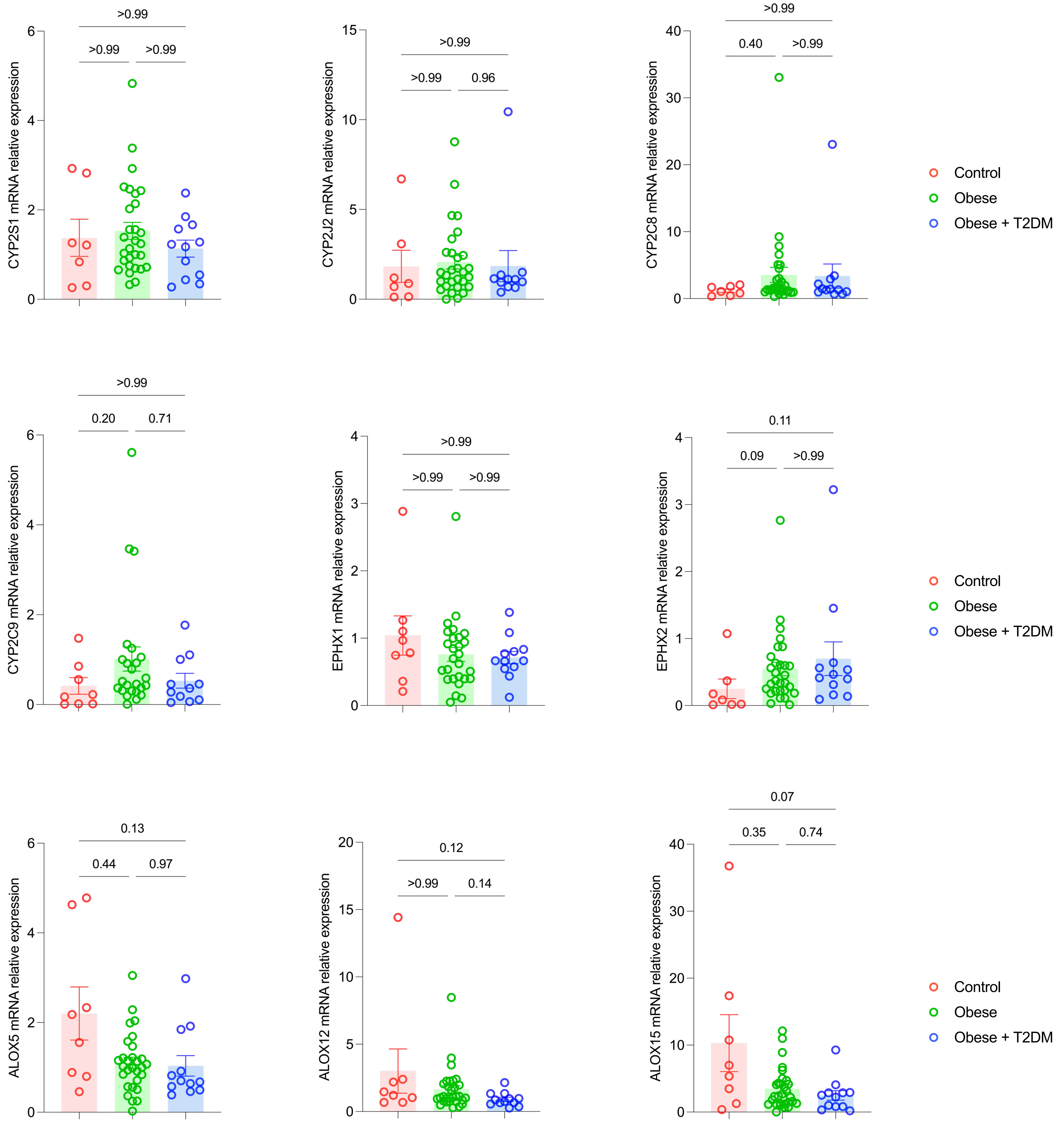

Supplementary Figure 4. **CYP, EPHX and ALOX enzyme expressions in lean, obese and obese/T2DM WAT of bariatric surgery patients**.

Expression of *CYP2S1*, *CYP2J2*, *CYP2C8*, *CYP2C9*, *EPHX1*, *EPHX2*, *ALOX5*, *ALOX12*, *ALOX15* in the WAT of lean (N=8), obese (N=24) and obese /T2DM (N=11). Statistical significance by Kruskal-Wallis with Dunn's multiple comparison test.
