# Supplementary material for: Multi-tissue profiling of oxylipins reveal a conserved up-regulation of epoxide:diol ratio that associates with white adipose tissue inflammation and liver steatosis in obesity
Source: eBioMedicine. 2024 Apr 26;103:105127. doi: 10.1016/j.ebiom.2024.105127 (PMC11061246; doi:10.1016/j.ebiom.2024.105127)
Supplement: Supplementary Figure 5 [file mmc5.pdf]

a

5,6-DHET	R	R^2	Beta	t	Sig.
	0.541	0.292			
Age			0.002	0.196	0.055
Sex [Female=1 male=2]			0.073	0.231	0.025
Ethnicity [white=1 black =2 mixed=3 asian=4 other=5]			0.015	-0.136	0.175
Obese[lean=1, obese=2]			0.104	-0.344	**0.003***
Total: 85					

11,12-DHET	R	R^2	Beta	t	Sig.
	0.459	0.211			
Age			0.002	0.113	0.291
Sex [Female=1 male=2]			0.063	0.294	0.007
Ethnicity [white=1 black =2 mixed=3 asian=4 other=5]			0.013	-0.165	0.12
Obese[lean=1, obese=2]			0.09	-0.232	**0.05***
Total: 85					

10,11-DIHDPA	R	R^2	Beta	t	Sig.
	0.506	0.256			
Age			0.002	0.208	0.062
Sex [Female=1 male=2]			0.079	0.018	0.873
Ethnicity [white=1 black =2 mixed=3 asian=4 other=5]			0.015	-0.196	0.079
Obese[lean=1, obese=2]			0.106	-0.402	**0.002***
Total: 85					

12,13-EpOME:DiHOME	R	R^2	Beta	t	Sig.
	0.299	0.089			
Age			0.002	-0.047	0.684
Sex [Female=1 male=2]			0.067	0.013	0.912
Ethnicity [white=1 black =2 mixed=3 asian=4 other=5]			0.013	0.082	0.468
Obese[lean=1, obese=2]			0.096	0.295	**0.021***
Total: 85					

b

5,6-DHET	R	R^2	Beta	t	Sig.
	0.341	0.116			
Age			0.002	0.035	0.764
Sex [Female=1 male=2]			0.071	-0.153	0.199
Ethnicity [white=1 black =2 mixed=3 asian=4 other=5]			0.014	0.043	0.704
Fasted [Fasted=1 non-Fasted=2]			0.059	0.246	0.055
Obese[lean=1, obese=2]			0.099	-0.152	0.285
Total: 82					

11,12-DHET	R	R^2	Beta	t	Sig.
	0.435	0.19			
Age			0.001	0.047	0.671
Sex [Female=1 male=2]			0.049	-0.177	0.122
Ethnicity [white=1 black =2 mixed=3 asian=4 other=5]			0.01	0.004	0.969
Fasted [Fasted=1 non-Fasted=2]			0.041	0.285	0.021
Obese[lean=1, obese=2]			0.069	-0.234	0.086
Total: 82					

10,11-DiHDPA	R	R^2	Beta	t	Sig.
	0.523	0.274			
Age			0.002	0.282	0.01
Sex [Female=1 male=2]			0.08	-0.228	0.039
Ethnicity [white=1 black =2 mixed=3 asian=4 other=5]			0.016	-0.076	0.464
Fasted [Fasted=1 non-Fasted=2]			0.068	0.07	0.551
Obese[lean=1, obese=2]			0.112	-0.34	**0.011***
Total: 82					

12,13-EpOME:DiHOME	R	R^2	Beta	t	Sig.
	0.466	0.217			
Age			0.002	0.003	0.98
Sex [Female=1 male=2]			0.089	0.199	0.077
Ethnicity [white=1 black =2 mixed=3 asian=4 other=5]			0.018	0.036	0.732
Fasted [Fasted=1 non-Fasted=2]			0.074	-0.289	0.018
Obese[lean=1, obese=2]			0.124	0.293	**0.03***
Total: 82					

Supplementary Figure 5. **Multivariate analysis of CYP-derived diols conserved between WAT and plasma**. Linear regression tables for CYP-derived diols and 12,13-EpOME:diHOME measured in WAT (**a**) and plasma (**b**), adjusted for age, sex and ethnicity; N=85 for WAT; N=82 for plasma. Statistical significance was calculated using a linear regression model with normalization of dependent variable by log transformation of the metabolite.
