# Supplementary material for: Multi-tissue profiling of oxylipins reveal a conserved up-regulation of epoxide:diol ratio that associates with white adipose tissue inflammation and liver steatosis in obesity
Source: eBioMedicine. 2024 Apr 26;103:105127. doi: 10.1016/j.ebiom.2024.105127 (PMC11061246; doi:10.1016/j.ebiom.2024.105127)
Supplement: Supplementary Figure 6 [file mmc6.pdf]

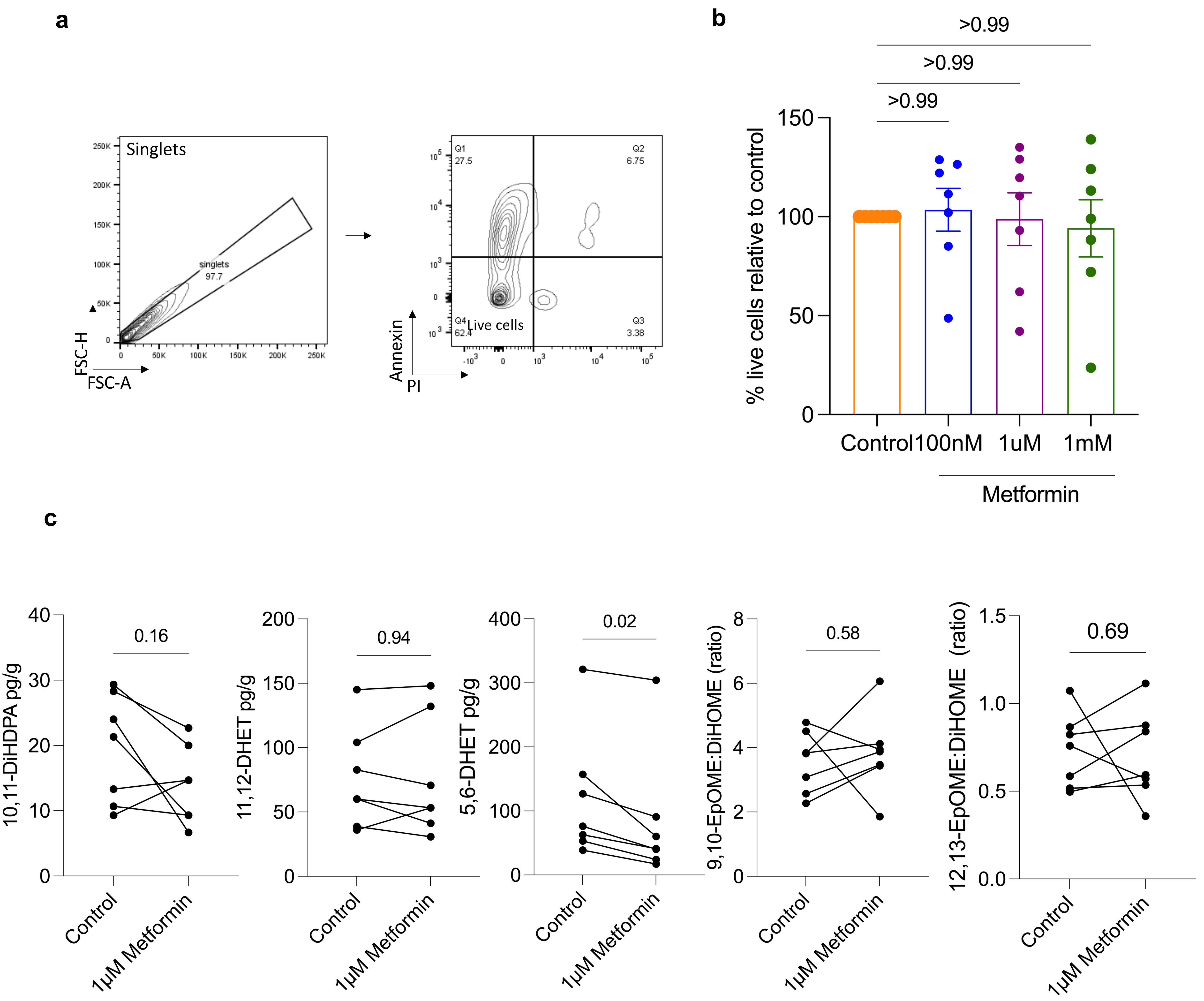

Supplementary Figure 6. ***Ex vivo* metformin effect on fatty acid diols and epoxide:diol in explants isolated from bariatric surgery patients**. (**a**) Gating strategy for quantifying number of viable cells (PI/Annexin staining) in the stromal vascular fraction (SVF) isolated from WAT explants (N=7). (**b**) Percentage SVF cell viability following incubation with Metformin (100nM, 1μM and1mM; 48h) compared to control (100%). (**c**) 10,11-DiHDPA, 11,12-DHET. 5,6-DHET, 9,10-EpOME:DiHOME and 12,13-EpOME:DiHOME levels in explants treated with or without 1μM Metformin (N=7). Statistical significance measure by Kruskal-Wallis with Dunn's multiple comparison test (**b**) and Wilcoxon matched pairs signed rank test (**c**).
