# Supplementary material for: Multi-tissue profiling of oxylipins reveal a conserved up-regulation of epoxide:diol ratio that associates with white adipose tissue inflammation and liver steatosis in obesity
Source: eBioMedicine. 2024 Apr 26;103:105127. doi: 10.1016/j.ebiom.2024.105127 (PMC11061246; doi:10.1016/j.ebiom.2024.105127)
Supplement: Supplementary Figure 7 [file mmc7.pdf]

a

Liver 14,15-EET:DHET (free)	R	R^2	Beta	t	Sig.
	0.551	0.303			
Sex [Female=1 male=2]			0.066	-0.014	0.932
Age			0.002	-0.173	0.347
Ethnicity [white=1 black =2 mixed=3 asian=4 other=5]			0.017	0.329	0.053
T2DM [y=1 n=2]			0.062	-0.483	***0.009****
Total 31					

b

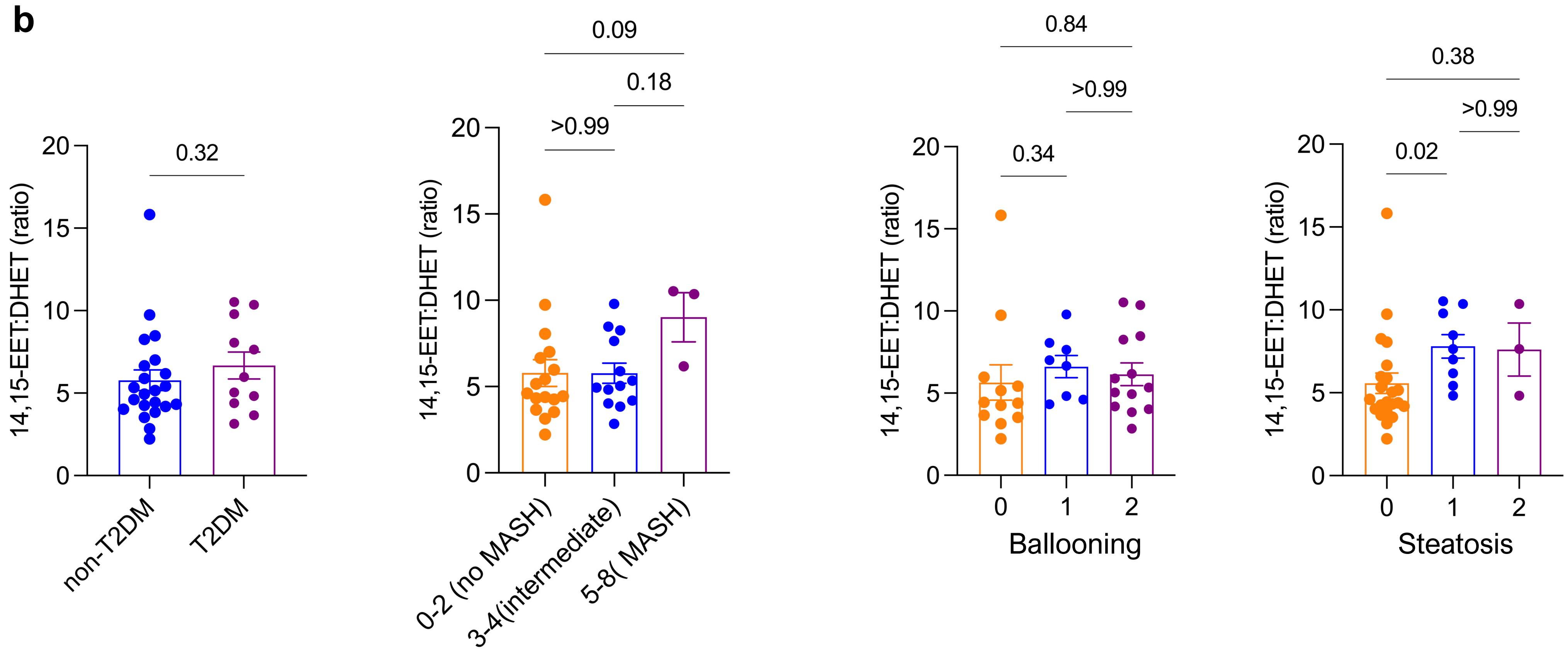

c

Liver 14,15-EET:DHET (total)	R	R^2	Beta	t	Sig.
	0.45	0.202			
Sex [Female=1 male=2]			1.156	-0.228	0.209
Age			0.04	-0.002	0.994
Ethnicity [white=1 black =2 mixed=3 asian=4 other=5]			0.299	0.395	0.032
T2DM [y=1 n=2]			1.093	-0.199	0.29
Total 31					

Supplementary Figure 7. **Multivariate analysis for hepatic 14,15-EET:DHET based on free (unesterified) and total epoxide:diol. (a)** Linear regression table for free 14,15-EET:DHET measured in liver adjusted for age, sex and ethnicity; N=31. (**b**) T2DM and MASLD phenotypes for total 14,15-EET:DHET. (**c**) Linear regression table for total 14,15-EET:DHET measured in liver adjusted for age, sex and ethnicity; N=31. Statistical significance was calculated using a linear regression model with normalization of dependent variable by log transformation of the metabolite (**a**) and (**c**) and Mann-Whitney test or Kruskal-Wallis test with Dunn's multiple comparisons test (**b**).
