# Supplementary material for: Multi-tissue profiling of oxylipins reveal a conserved up-regulation of epoxide:diol ratio that associates with white adipose tissue inflammation and liver steatosis in obesity
Source: eBioMedicine. 2024 Apr 26;103:105127. doi: 10.1016/j.ebiom.2024.105127 (PMC11061246; doi:10.1016/j.ebiom.2024.105127)
Supplement: Supplementary Figure 8 [file mmc8.pdf]

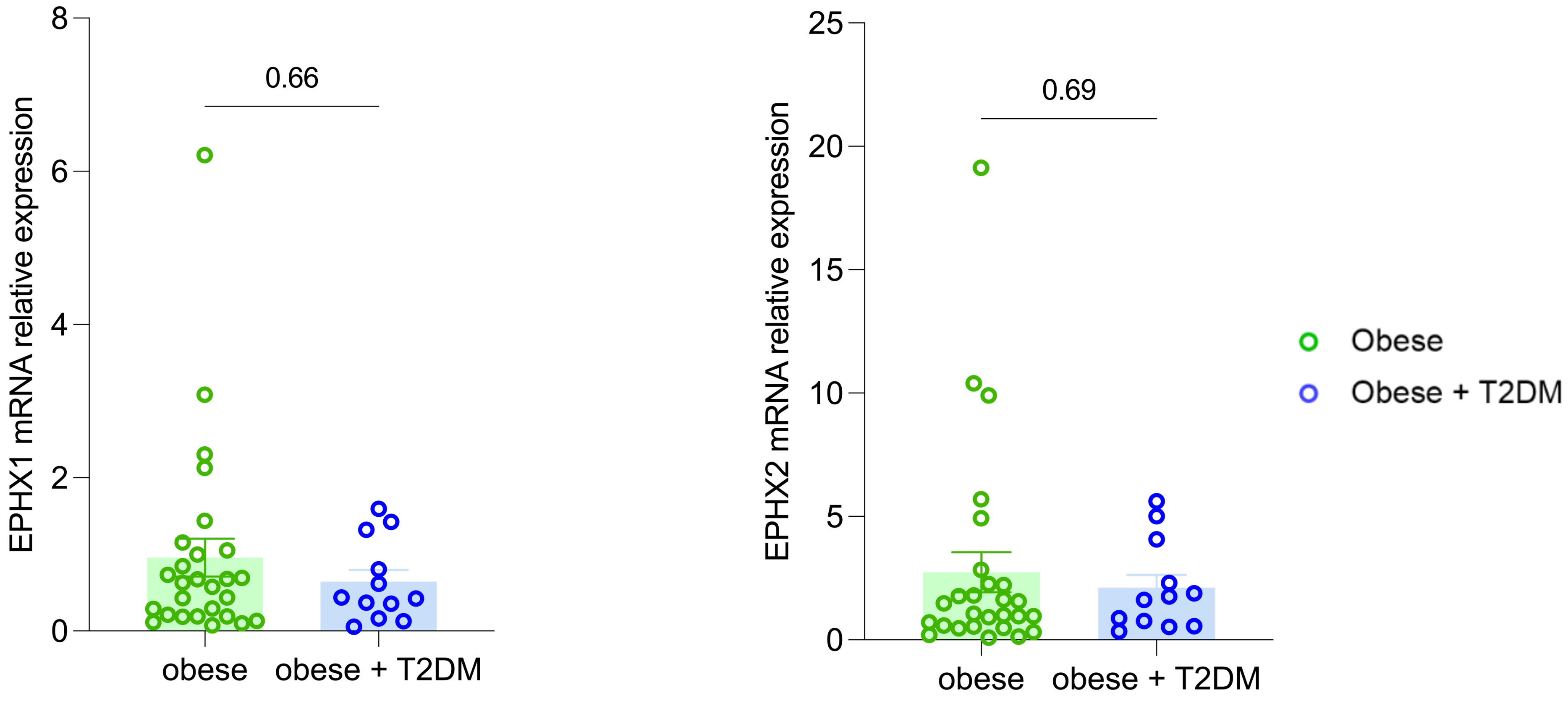

Supplementary Figure 8. **Hepatic *EPHX1* and *EPHX2* expression in obese patients with and without T2DM**. *EPHX1* and *EPHX2* in the liver of obese (N=27) and obese patients with T2DM (N=12). Statistical significance measured by Mann-Whitney test.
