# Supplementary material for: Multi-tissue profiling of oxylipins reveal a conserved up-regulation of epoxide:diol ratio that associates with white adipose tissue inflammation and liver steatosis in obesity
Source: eBioMedicine. 2024 Apr 26;103:105127. doi: 10.1016/j.ebiom.2024.105127 (PMC11061246; doi:10.1016/j.ebiom.2024.105127)
Supplement: Supplementary Figure 9 [file mmc9.pdf]

10,11-EpDPA:DiHDPA	R	R^2	Beta	t	Sig.
	0.676	0.457			
Sex [Female=1 male=2]			-0.029	-0.178	0.86
Age			-0.087	-0.49	0.629
Ethnicity [white=1 black =2 mixed=3 asian=4 other=5]			0.148	0.874	0.391
CPA (%)			-0.135	-0.818	0.421
Fat (%)			0.444	2.729	**0.012***
Ballooning (%)			0.438	2.47	**0.021***
Inflammation (%)			0.157	0.915	0.369
Total 31					

11,12-EET:DHET	R	R^2	Beta	t	Sig.
	0.674	0.454			
Sex [Female=1 male=2]			-0.044	-0.266	0.793
Age			0.099	0.557	0.582
Ethnicity [white=1 black =2 mixed=3 asian=4 other=5]			0.219	1.29	0.209
CPA (%)			0.116	0.701	0.49
Fat (%)			0.506	3.102	**0.005***
Ballooning (%)			0.424	2.384	**0.025***
Inflammation (%)			0.267	1.555	0.133
Total 31					

14,15-EET:DHET	R	R^2	Beta	t	Sig.
	0.678	0.46			
Sex [Female=1 male=2]			-0.042	-0.259	0.798
Age			0.065	0.37	0.715
Ethnicity [white=1 black =2 mixed=3 asian=4 other=5]			0.109	0.644	0.526
CPA (%)			-0.078	-0.472	0.641
Fat (%)			0.523	3.222	**0.004***
Ballooning (%)			0.466	2.636	**0.014***
Inflammation (%)			0.066	0.385	0.704
Total 31					

12,13-EpOME:DiHOME	R	R^2	Beta	t	Sig.
	0.61	0.372			
Sex [Female=1 male=2]			-0.08	-0.454	0.654
Age			0.017	0.091	0.928
Ethnicity [white=1 black =2 mixed=3 asian=4 other=5]			0.233	1.28	0.213
CPA (%)			0.293	1.647	0.112
Fat (%)			0.444	2.539	**0.018***
Ballooning (%)			0.263	1.381	0.18
Inflammation (%)			0.172	0.937	0.358
Total 31					

19,20-EPDPE:DiHDPA	R	R^2	Beta	t	Sig.
	0.519	0.27			
Sex [Female=1 male=2]			-0.184	-0.969	0.342
Age			-0.331	-1.615	0.119
Ethnicity [white=1 black =2 mixed=3 asian=4 other=5]			-0.115	-0.583	0.565
CPA (%)			0.164	0.856	0.4
Fat (%)			0.095	0.505	0.618
Ballooning (%)			0.107	0.523	0.606
Inflammation (%)			0.284	1.43	0.166
Total 31					

17,18-EpETE:DiHETE	R	R^2	Beta	t	Sig.
	0.471	0.222			
Sex [Female=1 male=2]			0.243	1.242	0.226
Age			0.309	1.458	0.158
Ethnicity [white=1 black =2 mixed=3 asian=4 other=5]			0.166	0.82	0.421
CPA (%)			0.026	0.13	0.897
Fat (%)			0.085	0.434	0.668
Ballooning (%)			0.292	1.378	0.181
Inflammation (%)			0.296	1.444	0.162
Total 31					

Supplementary Figure 9. **Multivariate analysis of different epoxide:diol measured in MASLD liver biopsies of bariatric surgery patients**. Linear regression tables for 10,11-EpDPA:DiHDPA, 11,12-EET:DHET, 14,15:EET:DHET, 12,13-EpOME:DiHOME, 19,20-EpDPE:DiHDPA, 17,18-EpETE:DiHETE in MASLD liver tissue adjusted for confounding factors (age, gender and ethnicity; N=31). Markers of MASLD were measured by AI which calculated percentage of CPA, ballooning, steatosis and inflammation in individual liver samples (see methods). Statistical significance was calculated using a linear regression model with normalization of dependent variable by log transformation of metabolite activity.
