# Supplementary material for: Multi-tissue profiling of oxylipins reveal a conserved up-regulation of epoxide:diol ratio that associates with white adipose tissue inflammation and liver steatosis in obesity
Source: eBioMedicine. 2024 Apr 26;103:105127. doi: 10.1016/j.ebiom.2024.105127 (PMC11061246; doi:10.1016/j.ebiom.2024.105127)
Supplement: Supplemental Western Blot File [file mmc10.pptx]

## Slide 1
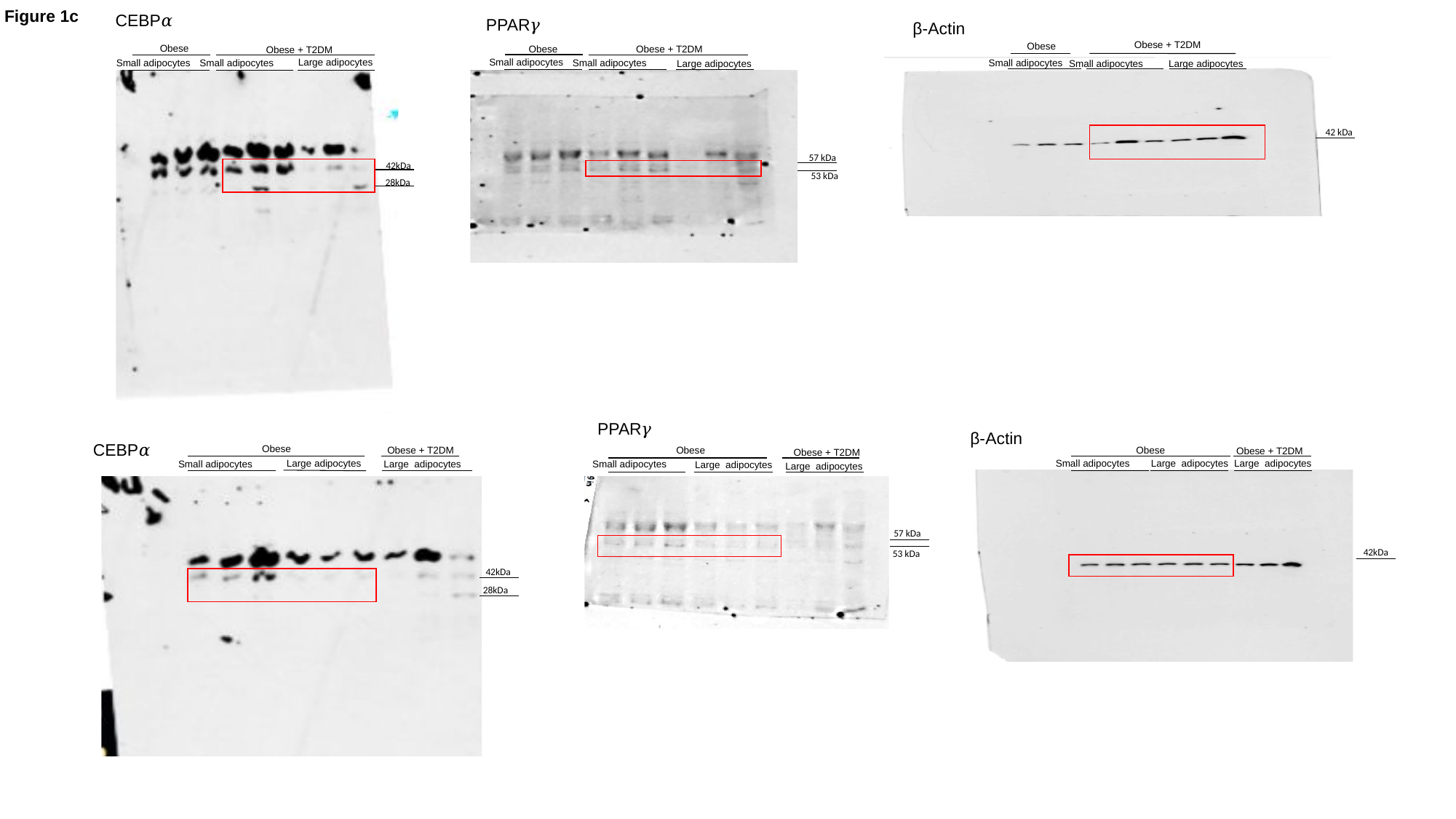

Figure 1c
CEBP𝛼
PPAR𝛾
β-Actin
Obese + T2DM
 Obese
Small adipocytes
Obese
Obese + T2DM
Small adipocytes
Obese + T2DM
Obese
Small adipocytes
Small adipocytes
Large adipocytes
Small adipocytes
Small adipocytes
Large adipocytes
Large adipocytes
42 kDa
57 kDa
42kDa
53 kDa
28kDa
PPAR𝛾
β-Actin
CEBP𝛼
Obese
Obese + T2DM
Obese
Obese
Obese + T2DM
Obese + T2DM
Large adipocytes
Large adipocytes
Small adipocytes
Large adipocytes
Small adipocytes
Large adipocytes
Small adipocytes
Large adipocytes
Large adipocytes
57 kDa
42kDa
53 kDa
42kDa
28kDa
