# Supplementary material for: Multi-tissue profiling of oxylipins reveal a conserved up-regulation of epoxide:diol ratio that associates with white adipose tissue inflammation and liver steatosis in obesity
Source: eBioMedicine. 2024 Apr 26;103:105127. doi: 10.1016/j.ebiom.2024.105127 (PMC11061246; doi:10.1016/j.ebiom.2024.105127)
Supplement: Supplementary Table 1 [file mmc11.pdf]

Human gene	Forward Primer (5'- 3')	Reverse Primer (3'-5')
COL1A1	TCCGACCTCTCTCCTCTGAA	GTCTTTTGCTTCCTCCCACC
COL6A1	CGCTTCATCGACAACCTGAG	TCCAGCCCCTTCTTGATAGC
CD68	CCTCCAAGCCCAGATTCAGA	CATTGTACTCCACCGCCATG
TREM2	TGCGGAATCTACAACCCAT	GCATCCTCGAAGCTCTCAGA
ALOX5	ATGCCAACAAAACAGACCCC	TGGAACGCACCCAGATTTTG
ALOX12	TCTCCAACCCCAACACTGTT	GGAACTTGAAGATGGGGTGC
ALOX15	CTGCAACTGGATCTCTGTGC	TCTTCCAGCTCTTCTTCCCG
CYP2S1	TGGGCATGGACAGGGTTAAT	GTTGCTTTGTGGCCCTTGTA
CYP2J2	GGAGAACGGACAGCCTTTTG	AGCTGGCATGTCTTTGAAGC
CYP2C8	TGACCCTGGCCACTTTCTAG	ATGAGGGTGGCAGAGAAACA
CYP2C9	GGGGCAGCTCTAACCTCTAG	CTGGGAGGCGGAGGTTATAG
EPHX1	ATGACTCTCCTGTGGGTCTG	GAGGAGATGATGGTGCCTGT
EPHX2	GAAAGATCAACCGCCCCATG	CCCACCTGACACGACTCTAT
ACTIN	ATGTGGATCAGCAAGCAGGA	CAATCAAAGTCCTCGGCCAC
HRPT	TGAGGATTTGGAAAGGGTGT	AATCCAGCAGGTCAGCAAAG

Supplementary Table 1. Human primer sequences used in for qRT-PCR
