# Supplementary material for: Multi-tissue profiling of oxylipins reveal a conserved up-regulation of epoxide:diol ratio that associates with white adipose tissue inflammation and liver steatosis in obesity
Source: eBioMedicine. 2024 Apr 26;103:105127. doi: 10.1016/j.ebiom.2024.105127 (PMC11061246; doi:10.1016/j.ebiom.2024.105127)
Supplement: Supplementary Table 2 [file mmc12.pdf]

Compound	Compound (abbreviation)	MS1 (m/z)	MS2 (m/z)	RT (min)	Low Standard (pg/ul)	High Standard (pg/ul)	ISTD used	r_2	Adipose S/N	Liver S/N
6-Keto-prostaglandin F1alpha	6-keto-PGF1a	369.2	163.1	2.39	0.1	120	PGE2-d9	0.998	1864	76
Thromboxane B2	TXB2	369.2	169.2	3.21	0.025	30	PGE2-d9	0.997	44	83
Prostaglandin B2	PGB2	333.2	175.2	5.21	0.1	120	PGE2-d9	0.996	48	42
Prostaglandin D2	PGD2	351.2	271.1	3.96	0.025	30	PGE2-d9	0.969	1025	519
Prostaglandin E2	PGE2	351.2	271.1	3.75	0.025	30	PGE2-d9	0.994	701	356
8-iso-prostaglandin F2 alpha	8-iso-PGF2a	353.2	193.1	3.21	0.05	60	PGE2-d9	0.995	45	BD
Prostaglandin F2alpha	PGF2a	353.2	193.1	3.65	0.05	60	PGE2-d9	0.996	47	69
Prostaglandin D3	PGD3	349.2	269.2	3.31	0.025	30	PGE2-d9	0.951	BD	57
Prostaglandin E3	PGE3	349.2	269.2	3.16	0.025	30	PGE2-d9	0.99	BD	47
11-dehydrothromboxane B2	11-dehydro-TXB2	367.2	161.3	3.73	0.025	30	PGE2-d9	0.967	BD	158
15-keto Prostaglandin E2	15-ketoPGE2	349.2	113.1	4.8	0.025	30	PGE2-d9	0.97	BD	BD
15-Keto Prostaglandin F2alpha	15-keto-PGF2a	351.2	219.2	3.85	0.025	30	PGE2-d9	0.998	BD	BD
19,20-dihydroxy-4Z,7Z,10Z,13Z,19Z-docosapentaenoic acid	19,20-DiHDPA	361.2	273.2	6.77	0.1	120	11,12-DHET-d11	0.998	14	143
16,17-dihydroxy-4Z,7Z,10Z,13Z,19Z-docosapentaenoic acid	16,17-DiHDPA	361.2	233.1	7.06	0.1	120	11,12-DHET-d11	0.998	16	1296
13,14-dihydroxy-4Z,7Z,10Z,16Z,19Z-docosapentaenoic acid	13,14-DiHDPA	361.2	193.1	7.18	0.1	120	11,12-DHET-d11	0.998	32	1477
10,11-dihydroxy-4Z,7Z,13Z,16Z,19Z-docosapentaenoic acid	10,11-DiHDPA	361.2	153.1	7.35	0.1	120	11,12-DHET-d11	0.996	18	1928
7,8-dihydroxy-4Z,10Z,13Z,16Z,19Z-docosapentaenoic acid	7,8-DiHDPA	361.2	189.1	7.75	0.1	120	11,12-DHET-d11	0.998	BD	170
7,18-dihydroxy-5Z,8Z,11Z,14Z-eicosatetraenoic acid	17,18-DiHETE	335.2	247.1	5.89	0.1	120	11,12-DHET-d11	0.999	24	1338
14,15-dihydroxy-5Z,8Z,11Z,17Z-eicosatetraenoic acid	14,15-DiHETE	335.2	207.1	6.13	0.1	120	11,12-DHET-d11	0.999	22	710
11,12-dihydroxy-5Z,8Z,14Z-eicosatrienoic acid	11,12-DiHETE	335.2	167.1	6.23	0.1	120	11,12-DHET-d11	0.978	BD	572
12,13-dihydroxy-9Z-octadecenoic acid	12,13-DiHOME	313.2	183.2	6.29	1	1200	11,12-DHET-d11	0.987	749	3435
9,10-dihydroxy-12Z-octadecenoic acid	9,10-DiHOME	313.2	201.1	6.5	1	1200	11,12-DHET-d11	0.97	3230	11700
14,15-dihydroxy-5Z,8Z,11Z-eicosatrienoic acid	14,15-DHET	337.2	207.2	6.75	0.1	120	11,12-DHET-d11	0.997	31	9736
11,12-dihydroxy-5Z,8Z,14Z-eicosatrienoic acid	11,12-DHET	337.2	167.2	7.09	0.1	120	11,12-DHET-d11	0.997	138	4927
8,9-dihydroxy-8Z,11Z,14Z-eicosatrienoic acid	8,9-DHET	337.2	127.3	7.4	0.1	120	11,12-DHET-d11	0.997	29	1019
5,6-dihydroxy-8Z,11Z,14Z-eicosatrienoic acid	5,6-DHET	337.2	145.1	7.84	0.1	120	11,12-DHET-d11	0.998	84	8540
19,20-epoxy-4Z,7Z,10Z,13Z,16Z-docosapentaenoic acid	19,20-EpDPE	343.2	281.1	10.34	0.2	240	11,12-EET-d11	0.998	53	160
16,17-epoxy-4Z,7Z,10Z,13Z,19Z-docosapentaenoic acid	16,17-EpDPE	343.2	233.1	10.78	0.2	240	11,12-EET-d11	0.998	BD	21
13,14-epoxy-4Z,7Z,10Z,16Z,19Z-docosapentaenoic acid	13,14-EpDPE	343.2	193.1	10.89	0.2	240	11,12-EET-d11	0.998	BD	54
10,11-epoxy-4Z,7Z,13Z,16Z,19Z-docosapentaenoic acid	10,11-EpDPE	343.2	153	10.98	0.2	240	11,12-EET-d11	0.997	BD	936
7,8-epoxy-4Z,10Z,13Z,16Z,19Z-docosapentaenoic acid	7,8-EpDPE	343.2	189.1	11.17	0.2	240	11,12-EET-d11	0.997	BD	205
17,18-epoxy-5Z,8Z,11Z,14Z-eicosatetraenoic acid	17,18-EpETE	317.2	259.1	8.94	0.1	120	11,12-EET-d11	0.998	BD	33
14,15-epoxy-5Z,8Z,11Z,17Z-eicosatetraenoic acid	14,15-EpETE	317.2	207.1	9.42	0.2	240	11,12-EET-d11	0.998	BD	110
11,12-epoxy-5Z,8Z,14Z,17Z-eicosatetraenoic acid	11,12-EpETE	317.2	167.1	9.5	0.2	240	11,12-EET-d11	0.978	BD	45
12,13-epoxy-9Z-octadecenoic acid	12,13-EpOME	295.2	195.2	10.34	0.5	600	11,12-EET-d11	0.984	1568	1695
9,10-epoxy-12Z-octadecenoic acid	9,10-EpOME	295.2	277.3	10.54	0.5	600	11,12-EET-d11	0.991	418	457
14,15-epoxy-5Z,8Z,14Z-eicosatrienoic acid	14,15-EET	319.2	219.3	10.52	0.2	240	11,12-EET-d11	0.998	73	262
11,12-epoxy-5Z,8Z,14Z-eicosatrienoic acid	11,12-EET	319.2	179.2	10.9	0.2	240	11,12-EET-d11	0.997	71	129
8,9-epoxy-5Z,11Z,14Z-eicosatrienoic acid	8,9-EET	319.2	151.1	11	0.2	240	11,12-EET-d11	0.996	22	81
5,6-epoxy-8Z,11Z,14Z-eicosatrienoic acid	5,6-EET	319.2	191.3	11.17	0.2	240	11,12-EET-d11	NA	BD	127
20-hydroxydocosa-4Z,7Z,10Z,13Z,16Z,18Z-hexaenoic acid	22-HDoHE	343.2	269.1	8.07	0.025	30	d8-15-HETE	0.997	BD	249
17-hydroxy-4Z,7Z,10Z,13Z,15E,19Z-docosahexaenoic acid,	17-HDHA	343.2	281.2	9.04	0.025	30	d8-15-HETE	0.999	1872	197
12-hydroxy-5Z,8Z,10E,14Z,17Z-eicosapentaenoic acid	20-HEPE	317.2	243.2	7.08	0.025	30	d8-15-HETE	0.998	BD	27
18-hydroxyeicosa-5Z,8Z,11E,14Z,16E-pentaenoic acid	18-HEPE	317.2	161.4	7.27	0.025	30	d8-15-HETE	0.998	BD	28
13-hydroxy-9Z,11E-octadecadienoic acid	13-HODE	295.2	195.2	8.47	2.5	3000	d8-15-HETE	0.967	63600	7140
9-hydroxy-10E,12Z-octadecadienoic acid	9-HODE	295.2	171.2	8.59	2.5	3000	d8-15-HETE	0.974	11700	15700
20-hydroxy-5Z,8Z,11Z,14Z-eicosatetraenoic acid	20-HETE	319.2	245.1	7.8	0.5	600	d8-15-HETE	0.99	BD	91
19-hydroxy-5Z,8Z,11Z,14Z-eicosatetraenoic acid	19-HETE	319.2	231.1	7.68	0.5	600	d8-15-HETE	0.987	BD	399
15-hydroxy-5Z,8Z,11Z,13E-eicosatetraenoic acid	15-HETE	319.2	219.1	8.87	0.1	120	d8-15-HETE	0.996	37900	1494
12-hydroxy-5Z,8Z,10E,14Z-eicosatetraenoic acid	12-HETE	319.2	179.2	9.47	1.25	1500	d8-15-HETE	0.973	6710	4105
11-hydroxy-5E,8Z,12Z,14Z-eicosatetraenoic acid	11-HETE	319.2	167.2	9.22	0.1	120	d8-15-HETE	0.998	4269	1744
8-hydroxy-5Z,9E,11Z,14Z-eicosatetraenoic acid	8-HETE	319.2	163.3	9.47	0.1	120	d8-15-HETE	9.63	407	167
5S-hydroxy-6e,8Z,11Z,14Z-eicosatetraenoic acid	5-HETE	319.2	257.3	9.47	0.1	120	d8-15-HETE	0.997	2236	1603
Lipoxin A4	Lipoxin_A4	351.2	115.1	4.43	0.025	30	d8-15-HETE	0.996	90	30
Lipoxin B4	Lipoxin_B4	351.2	221.1	3.96	0.025	30	d8-15-HETE	0.998	27	BD
Leukotriene B4	LTB4	335.2	195.3	6.11	0.025	30	d4-LTB4	0.998	38	BD
20-carboxy arachidonic acid	20-carboxy-AA	333.2	271.2	7.31	0.025	30	d4-LTB4	0.99	252	123
Docosahexaenoic acid	DHA	327.2	283.2	12.69	15	18000	AA-d8	0.989	46583	78500
Eicosapentaenoic acid	EPA	301.2	257.2	12.28	15	18000	AA-d8	0.979	41750	121800
Linoleic acid	LA	279.2	261.2	12.86	15	18000	AA-d8	0.981	21900	124900
Arachidonic acid	AA	303.2	259.2	12.8	15	18000	AA-d8	0.978	103800	114900
Prostaglandin E2-d9	PGE2-d9	360.2	280.1	3.72						
11,12-epoxy-5Z,8Z,14Z-eicosatrienoic acid-d11	11,12-EET-d11	330.2	179.2	10.9						
11,12-dihydroxy-5Z,8Z,14Z-eicosatrienoic acid-d11	11,12-DHET-d11	348.3	167.1	7.02						
15-hydroxyeicosatetraenoic acid-d8	d8-15-HETE	327.2	226.1	8.77						
d4-leukotriene B4	d4-LTB4	339.2	197.1	6.08						
Arachidonic acid-d8	AA-d8	311.2	267.2	12.79						

Supplementary Table 2. **Reference standards and internal standards used to assess oxylipin concentrations.** Ionization masses (MS1 and MS2) in mass/charge ratio (m/z), retention time (RT), the minimum/maximum concentrations used in the standard curve (pg/ul injected) and reference internal standard are indicated. Analyte quantification was determined using standard curves determined based on indicated ISTDs. r^2 values of the derived standard curves are shown. Average signal to noise (S/N) ratios for adipose tissue and liver (free) are also shown.
