# Supplementary material for: Multi-tissue profiling of oxylipins reveal a conserved up-regulation of epoxide:diol ratio that associates with white adipose tissue inflammation and liver steatosis in obesity
Source: eBioMedicine. 2024 Apr 26;103:105127. doi: 10.1016/j.ebiom.2024.105127 (PMC11061246; doi:10.1016/j.ebiom.2024.105127)
Supplement: Supplementary Table 3 [file mmc13.pdf]

oxylipin	WAT		Plasma		oxylipin	WAT		Plasma	
	p-value	FDR	p-value	FDR		p-value	FDR	p-value	FDR
20-carboxy-AA	0.77	0.87	0.09	0.15	5-HETE	0.66	0.82	0.51	0.57
AA	0.81	0.90	0.14	0.22	8-HETE	0.43	0.71	0.81	0.83
DHA	0.74	0.86	0.17	0.26	11-HETE	0.58	0.78	0.93	0.93
EPA	0.93	0.97	0.62	0.66	12-HETE	0.51	0.74	0.008*	0.037
Linoleic acid	0.31	0.56	0.015*	0.055	15-HETE	0.28	0.53	0.051	0.10
10,11-EpDPA	0.54	0.74	0.0015*	0.020	19-HETE	n.d.	n.d.	0.50	0.57
13,14-EpDPA	0.52	0.74	n.d.	n.d.	20-HETE	n.d.	n.d.	0.045*	0.10
16,17-EpDPA	0.12	0.29	n.d.	n.d.	Lipoxin_A4	0.12	0.29	n.d.	n.d.
19,20-EpDPE	0.06	0.22	0.287	0.38	Lipoxin_B4	0.13	0.30	n.d.	n.d.
7,8-DiHDPA	n.d.	n.d.	0.004*	0.023	9-HODE	0.10	0.28	0.49	0.57
10,11-DiHDPA	0.014*	0.11	0.049*	0.10	13-HODE	0.046*	0.21	0.28	0.38
13,14-DiHDPA	n.d.	n.d.	0.004*	0.023	17-HDHA	0.1	0.28	0.16	0.25
16,17-DiHDPA	n.d.	n.d.	0.014*	0.055	22-HDoHE	n.d.	n.d.	0.015*	0.055
19,20-DiHDPA	0.39	0.69	0.001*	0.017	18-HEPE	n.d.	n.d.	0.27	0.37
17,18-EpETE	n.d.	n.d.	0.019*	0.057	PGB2	0.14	0.31	0.008*	0.037
18-HEPE	0.46	0.73	n.d.	n.d.	PGD2	0.74	0.86	0.017	0.057
11,12-DiHETE	n.d.	n.d.	0.043*	0.10	PGE2	0.86	0.93	0.001*	0.017
14,15-DiHETE	0.07	0.24	0.18	0.26	15ketoPGE2	0.60	0.78	n.d.	n.d.
17,18-DiHETE	0.15	0.32	0.08	0.14	6ketoPGF1a	0.89	0.95	n.d.	n.d.
8,9-EET	0.06	0.22	<0.0001*	0.005	PGF2a	0.95	0.97	0.22	0.31
11,12-EET	0.11	0.29	0.05	0.10	8isoPGF2a	0.54	0.74	0.021*	0.059
14,15-EET	0.61	0.78	0.55	0.60	TXB2	0.51	0.74	0.002*	0.020
5,6-DHET	<.001*	0.026	0.023*	0.059	11-dehydro-TXB2	0.41	0.70	n.d.	n.d.
8,9-DHET	0.018*	0.11	0.07	0.13	10,11-EpDPA:DiHDPA	0.22	0.43	0.003*	0.023
11,12-DHET	0.018*	0.11	0.004*	0.023	19,20EpDPE:DiHDPA	0.047*	0.21	0.047*	0.10
14,15-DHET	0.013*	0.11	0.07	0.13	8,9-EET:DHET	1.00	1.00	0.022*	0.059
9,10-EpOME	0.18	0.37	0.34	0.41	11,12-EET:DHET	0.74	0.86	0.34	0.41
12,13-EpOME	0.046*	0.21	0.42	0.50	14,15-EET:DHET	0.09	0.28	0.81	0.83
9,10-DHOME	<.001*	0.026	0.08	0.14	9,10-EpOME:DiHOME	0.006*	0.077	0.333	0.41
12,13-DHOME	0.002*	0.034	n.d.	n.d.	12,13-EpOME:DiHOME	0.026*	0.15	0.019*	0.057

Supplementary Table 3. WAT and plasma oxylipin quantification. Oxylipins were quantified in control (N=7), obese (N=52) and obese with T2DM patients (N=26). All significant metabolites decreased while epoxide:diol ratios increased with obesity and T2DM, except for 10,11-EpDPA:DiHDPA. Statistical significance was tested between the three groups using Kruskal-Wallis test; False Discovery Rate (FDR) calculated using Benjamini-Hochberg; n.d., not detected.
