# Supplementary material for: Multi-tissue profiling of oxylipins reveal a conserved up-regulation of epoxide:diol ratio that associates with white adipose tissue inflammation and liver steatosis in obesity
Source: eBioMedicine. 2024 Apr 26;103:105127. doi: 10.1016/j.ebiom.2024.105127 (PMC11061246; doi:10.1016/j.ebiom.2024.105127)
Supplement: Certicate of compliance Type VI collagen [file mmc14.pdf]

abcam

Certificate of Compliance

**Product code**	ab199720
**Lot number**	GR3264378
**Product name**	Anti-Collagen VI antibody [EPR17077] - C-terminal

**Description**	Rabbit monoclonal [EPR17077] to Collagen VI - C-terminal
**Specificity**	Collagen VI
**Tested applications**	**Suitable for:** IHC-P, WB
**Species reactivity**	**Reacts with:** Mouse, Rat, Human
**Immunogen**	Recombinant fragment. This information is proprietary to Abcam and/or its suppliers.

**Form**	Liquid
**Storage instructions**	Shipped at 4°C. Store at +4°C short term (1-2 weeks). Upon delivery aliquot. Store at -20°C long term. Avoid freeze / thaw cycle.
**Storage buffer**	pH: 7.2<br>Preservative: 0.01% Sodium azide<br>Constituents: 59% PBS, 40% Glycerol (glycerin, glycerine), 0.05% BSA
**Concentration**	0.951 mg/ml
**Purity**	Protein A purified
**Isotype**	IgG

Abcam warrants that this product conforms to the information contained in this certificate.

The product shelf life is 12 months from date of receipt.

All our products are covered for 12 months by our Abpromise guarantee.

For more information, please visit **www.abcam.com/abpromise**.

Date Issued 29 January 2024

All products are **for research use only and are not intended for diagnostic or therapeutic use, not for use in humans.**
