# Supplementary material for: Multi-tissue profiling of oxylipins reveal a conserved up-regulation of epoxide:diol ratio that associates with white adipose tissue inflammation and liver steatosis in obesity
Source: eBioMedicine. 2024 Apr 26;103:105127. doi: 10.1016/j.ebiom.2024.105127 (PMC11061246; doi:10.1016/j.ebiom.2024.105127)
Supplement: Certificate of Analysis CD206 [file mmc16.pdf]

Enabling Legendary Discovery™

## Certificate of Analysis

# Brilliant Violet 421™ anti-human CD206 (MMR)

Catalog No. 321125

Lot No. B255124

| | |
|---|---|
| **Clone:** | 15-2 |
| **Format:** | Brilliant Violet 421™ |
| **Isotype:** | Mouse IgG1, κ |
| **Concentration:** | 100 µg/mL |
| **Volume:** | 125 µL |
| **Applications:** | FC - Quality tested |
| **Storage:** | The antibody solution should be stored undiluted between 2°C and 8°C, and protected from prolonged exposure to light. **Do not freeze.** |
| **Formulation:** | Phosphate-buffered solution, pH 7.2, containing 0.09% sodium azide and BSA (origin USA). |
| **Preparation:** | The antibody was purified by affinity chromatography and conjugated with Brilliant Violet 421™ under optimal conditions. |
| **Expiration Date:** | January 31, 2021 |

This product lot has passed BioLegend's QC testing and is certified for use. For details on QC testing view our page at biolegend.com/en-us/quality-control.

*BioLegend guarantees the performance of this product until the expiration date. The guarantee is dependent upon proper storage and handling as instructed on our Product Data Sheets. Every lot of product is quality tested against a "gold standard" reference lot. A new lot is only released based on our defined QC specifications to ensure lot to lot reproducibility and reliability. BioLegend guarantees the stability and performance of all our products shipped at room temperature;

For research use only. Not for diagnostic use. BioLegend will not be held responsible for patent infringement or other violations that may occur with the use of our products.
BioLegend Inc., 8999 BioLegend Way, San Diego, CA 92121;
https://www.biolegend.com
