# Supplementary material for: Multi-tissue profiling of oxylipins reveal a conserved up-regulation of epoxide:diol ratio that associates with white adipose tissue inflammation and liver steatosis in obesity
Source: eBioMedicine. 2024 Apr 26;103:105127. doi: 10.1016/j.ebiom.2024.105127 (PMC11061246; doi:10.1016/j.ebiom.2024.105127)
Supplement: Certificate of Analysis CD68 [file mmc19.pdf]

CERTIFICATE OF ANALYSIS

Product Information Datasheet

MOUSE ANTI HUMAN CD68

(MCA5709)

BATCH NUMBER 153311

**Description:**	MOUSE ANTI HUMAN CD68
**Specificity:**	CD68
**Other names:**	MACROSIALIN
**Format:**	Purified
**Product Type:**	Monoclonal Antibody
**Clone:**	KP1
**Isotype:**	IgG1
**Quantity:**	0.2 mg

Applications

This product has been reported to work in the following applications. This information is derived from testing within our laboratories, peer-reviewed publications or personal communications from the originators. Please refer to references indicated for further information. For general protocol recommendations, please visit www.bio-rad-antibodies.com/protocols.

	Yes	No	Not Determined	Suggested Dilution
Flow Cytometry (1)	▪			
Immunohistology - Frozen			▪	
Immunohistology - Paraffin (2)	▪			
ELISA			▪	
Immunoprecipitation	▪			
Western Blotting	▪			
Immunofluorescence	▪			

Where this antibody has not been tested for use in a particular technique this does not necessarily exclude its use in such procedures. Suggested working dilutions are given as a guide only. It is recommended that the user titrates the antibody for use in their own system using appropriate negative/positive controls.

(1)**Membrane permeabilisation is required for this application. Leucoperm™ (BUF09A) is recommentded for this purpose.**

(2)**This antibody requires heat-mediated antigen retrieval prior to staining paraffin sections. Citrate buffer, pH6.0, is recommended for this purpose**.

**Target Species**	Human
**Product Form**	Purified IgG - liquid
**Preparation**	Purified IgG prepared by affinity chromatography on Protein A from tissue culture supernatant
**Buffer Solution**	Phosphate buffered saline
**Preservative Stabilisers**	0.09% Sodium Azide (NaN_3)
**Approx. Protein Concentrations**	IgG concentration 1.0mg/ml

**Flow Cytometry**	Use 10ul of the suggested working dilution to label 10^6 cells in 100ul.
**Storage**	Store at +4°C or at -20°C if preferred.<br><br>This product should be stored undiluted.<br><br>Storage in frost free freezers is not recommended. Avoid repeated freezing and thawing as this may denature the antibody. Should this product contain a precipitate we recommend microcentrifugation before use.
**Guarantee**	12 months from date of despatch
**Health And Safety Information**	Material Safety Datasheet documentation #10040 available at:<br>https://www.bio-rad-antibodies.com/SDS/MCA5709<br>10040
**Regulatory**	For research purposes only

Dr Anne-Sophie Rouziere
Technical Services Advisor

Tuesday, January 30, 2024

North & South America Tel: +1 800 265 7376
Fax: +1 919 878 3751
Email: antibody_sales_us@bio-rad.com

Worldwide Tel: +44 (0)1865 852 700
Fax: +44 (0)1865 852 739
Email: antibody_sales_uk@bio-rad.com

Europe Tel: +49 (0) 89 8090 95 21
Fax: +49 (0) 89 8090 95 50
Email: antibody_sales_de@bio-rad.com

To find a batch/lot specific datasheet for this product, please use our online search tool at: bio-rad-antibodies.com/datasheets

'M368336:200529'

Printed on 30 Jan 2024

© 2024 Bio-Rad Laboratories Inc | Legal | Imprint
