# Supplementary material for: Multi-tissue profiling of oxylipins reveal a conserved up-regulation of epoxide:diol ratio that associates with white adipose tissue inflammation and liver steatosis in obesity
Source: eBioMedicine. 2024 Apr 26;103:105127. doi: 10.1016/j.ebiom.2024.105127 (PMC11061246; doi:10.1016/j.ebiom.2024.105127)
Supplement: Certificate of Analysis CEBPA [file mmc20.pdf]

Cell Signaling TECHNOLOGY™

Certificate of Analysis

This document certifies that this product has met all of the quality control standards defined by Cell Signaling Technology, Inc.

Product Number: # **2295**

Product Name: **C/EBPα Antibody**

Product Type: Antibody

Species of Origin: Rabbit Polyclonal

Lot number: **#4**

Concentration: **28 ug/ml**

Approved Applications: (Check those that apply)

- [x] Western
- [ ] Immunoprecipitation
- [ ] Flow cytometry
- [ ] Immunohistochemistry
- [x] Immunofluorescence
- [ ] Peptide ELISA
- [ ] Chromatin Immunoprecipitation (ChIP)
- [ ] Carrier-free Custom Formulation
- [ ] Other (specify): _____

Approval:

Production Lead: Kathryn Abell, Group Leader

Signature: [signature] Date: 17 NOV 2014

Supervisor: Valerie Goss Ph.D, Senior Group Leader

Signature: [signature] Date: 17 NOV 2014

3 Trask Lane, Danvers, MA 01923
www.cellsignal.com

t 978-867-2300
f 978-867-2400
