# Supplementary material for: Multi-tissue profiling of oxylipins reveal a conserved up-regulation of epoxide:diol ratio that associates with white adipose tissue inflammation and liver steatosis in obesity
Source: eBioMedicine. 2024 Apr 26;103:105127. doi: 10.1016/j.ebiom.2024.105127 (PMC11061246; doi:10.1016/j.ebiom.2024.105127)
Supplement: Certificate of Analysis Collagen I [file mmc21.pdf]

SouthernBiotech

CERTIFICATE OF ANALYSIS

**Cat. No.**	1310-01
**Lot No.**	B2918-ZI20
**Description**	Goat Anti-Type I Collagen-UNLB
**Clone**	Polyclonal
**Concentration**	0.4 mg/mL
**Volume**	0.5 mL
**Expiration Date**	March 2022
**Storage**	2-8°C
**Buffer Formulation**	Borate buffered saline, pH 8.2
**Country of Origin**	USA

This document certifies that this product has met the quality control standards defined by SouthernBiotech and is guaranteed through the expiration date when stored undiluted as directed.

Certified By Lara Self

Quality Control

Certified Date 28Dec20

For Research Use Only. Not for Diagnostic or Therapeutic Use.

Corporate Offices: 160 Oxmoor Blvd • Birmingham, AL 35209 • USA **Mailing Address:** P.O. Box 26221 • Birmingham, AL 35260 • USA
Tel: 205.945.1774 • U.S. and Canada: 800.722.2255 • **Fax:** 205.945.1774
Email: info@southernbiotech.com • **Website:** www.southernbiotech.com
