# Supplementary material for: Multi-tissue profiling of oxylipins reveal a conserved up-regulation of epoxide:diol ratio that associates with white adipose tissue inflammation and liver steatosis in obesity
Source: eBioMedicine. 2024 Apr 26;103:105127. doi: 10.1016/j.ebiom.2024.105127 (PMC11061246; doi:10.1016/j.ebiom.2024.105127)
Supplement: Certificate of Analysis PPARY [file mmc22.pdf]

Cell Signaling TECHNOLOGY®

Certificate of Analysis

This document certifies that this product has met all of the quality control standards defined by Cell Signaling Technology, Inc.

Research Use Only Reagent (RUO): For research use only. Not for use in diagnostic procedures.

Product Number: **2435**
Product Name: **PPARγ (C26H12) Rabbit mAb**

Product Type: Monoclonal Antibody
Species of Origin: Rabbit
Lot Number: 7
Concentration: 125 µg/ml

Approved Applications:

- Chromatin IP
- Chromatin IP-seq
- Immunofluorescence (Immunocytochemistry)
- Immunohistochemistry (Paraffin)
- Western Blotting

Approval:	
Production: Valerie Goss	Date: 01-March-2021

3 Trask Lane, Danvers, MA 01923
www.cellsignal.com
phone: 978-867-2300
fax: 978-867-2400
